# Selected Abstracts from the 3rd European Hereditary Tumour Group Meeting (EHTG 2018): Nice, France. 23-25 September 2018

**DOI:** 10.1186/s13053-019-0115-7

**Published:** 2019-07-04

## N1 The German HNPCC consortium: aims, structure, methods and data

### C. Engel^1^, S. Aretz^2^

#### ^1^Institute for Medical Informatics, Statistics and Epidemiology, University of Leipzig, Leipzig, Germany; ^2^Institute of Human Genetics, Center for Hereditary Tumor Syndromes, Biomedical Center, University Hospital Bonn, Bonn, Germany

**Correspondence:** C. Engel

**Aim**

The German HNPCC Consortium, founded in 1999, is a joint network of currently 14 clinical university centers, a reference pathology, and a central documentation facility aiming to provide structured interdisciplinary care and research for individuals suspected of having an inherited predisposition for colorectal cancer. In the past, the consortium has focused on Lynch Syndrome (LS) but aims to cover also the broad spectrum of other familial colorectal cancer entities.

**Method**

Families are ascertained based on the established Amsterdam and Bethesda criteria. Interdisciplinary care comprises genetic counseling, molecular pathological tumour analyses for mismatch repair deficiency, germline testing of predisposing genes, and structured intensified surveillance measures. Research goals are e.g. search for new disease causing genes, genotype-phenotype correlations and tumour risks, tumour immunology, and efficacy of intensified surveillance.

**Results**

The consortium has established a central research database, which is populated by the clinical centres using a web-based remote online data capture application based on standardized documentation. The scope of the retro- and prospective data collection comprises fully structured pedigrees, familial tumour history, detailed results of diagnostics and results of surveillance.

**Conclusion**

Currently, approx. 8,800 individuals (patients, asymptomatic mutation carriers, relatives at risk) from 5,500 families are centrally registered (2,100 LS patients).

**Acknowledgements**

On behalf of the German HNPCC Consortium

## N3 Prevalence, phenotype and clinical consequences of mosaicism in APC and other colorectal cancer and polyposis associated genes

### M. Suerink^1^, S. Aretz^2,3^, A. Wagner^4^, M. Nielsen^1^, T. van Wezel^5^, H. Morreau^5^

#### ^1^Department of clinical genetics, LUMC, Leiden, The Netherlands; ^2^Institute of Human Genetics, University of Bonn, 53127 Bonn, Germany; ^3^Center for Hereditary Tumor Syndromes, University of Bonn, 53127 Bonn, Germany; ^4^Department of clinical genetics, Erasmuc Medical Centre, Rotterdam, The Netherlands; ^5^Department of pathology, LUMC, Leiden, The Netherlands

**Correspondence:** M. Suerink

**Aim**

APC mosaicism is identified in ~25% of previously unexplained polyposis patients with >20 adenomas. Prevalence of APC mosaicism in more mildly affected polyposis patients is currently unknown. Furthermore, surveillance advice is now the same for germline and mosaic APC patients, while a milder phenotype in the latter is expected. In the LUMC in Leiden, The Netherlands, a new study will start this year examining the prevalence of APC mosaic mutations in mildly affected polyposis patients as defined below. All mosaic patients will be recorded meticulously to determine phenotype.

**Method**

FFPE material of colorectal neoplasms (adenomas and/or colorectal cancers) of patients meeting the following criteria will be collected:

- >5 adenomas and aged <50
- >10 adenomas and aged <70y
- >20 adenomas and aged >70y
- meta- or synchronous CRC <70y
- 10-20 adenomas, between ages 55-75y, identified by population-based screening

We expect inclusion to start in the second half of 2018. DNA will be isolated from the neoplasms (n≥2) and a gene panel (including the following genes: APC, POLE/D1, MUTYH, NTHL1, MSH3, MLH1, MSH2, MSH6, PMS2, SMAD4, BMPR1A, ENG, RNF43, STK11, TP53, BRCA1, BRCA2, PALB2 and PTEN) will be run to identify APC mosaic cases as well as other (mosaic) causes of polyposis/colorectal cancer. Identification of the same mutation in multiple samples of the same patient will be considered to be indicative of mosaicism. Whenever possible, DNA isolated from leucocytes, buccal mucosa and urine will then be analyzed to see whether the variant can be identified in these tissues as well. Eligible patients will need to provide written consent before they are included.

**Results**

The main outcome will be prevalence of mosaic mutations in the above mentioned patient groups. Furthermore, mutation patterns and clinical phenotype will be recorded to study the mechanisms behind mosaicism and provide data to adapt surveillance guidelines.

**Conclusion**

A new study is starting this year at the LUMC in Leiden with the aim of further clarifying the prevalence, phenotype and clinical consequences of APC mosaicism. We invite attendees of the EHTG who have several cases that meet the selection criteria to contact us to discuss participation. We require tissue from multiple tumors from well described cases and can accommodate the NGS gene panel.

## N4 Breast cancer pathology and stage are better predicted by risk stratification models including mammographic density and common genetic variants

### D. G. Evans^1, 2, 6, 7, 8^, E. Harkness^2, 3, 4^, A. Brentnall^5^, E. van Veen^1^, S. Astley^2, 3, 4, 8^, H. Byers^1^, S. Sampson^2^, J. Southworth^2^, P. Stavrinos^2^, S. Howell^2, 6, 8^, A. Maxwell^2, 3, 4, 8^, A. Howell^2, 6, 8^, W. Newman^1,7,8^, J. Cuzick^5^

#### ^1^Division of Evolution and Genomic Sciences, Faculty of Biology, Medicine and Health, University of Manchester, Manchester, UK; ^2^Prevention Breast Cancer Unit and Nightingale Breast Screening Centre, Manchester University NHS Foundation Trust (South), Manchester, UK; ^3^Division of Informatics, Imaging and Data Sciences, School of Health Sciences, Faculty of Biology, Medicine and Health, University of Manchester, Manchester, UK; ^4^Manchester Academic Health Science Centre, University of Manchester, Manchester, UK; ^5^Centre for Cancer Prevention, Wolfson Institute of Preventive Medicine, Charterhouse Square, Barts and The London, Queen Mary University of London, London, UK; ^6^The Christie NHS Foundation Trust, Manchester, UK; ^7^Manchester Centre for Genomic Medicine, Manchester University NHS Foundation Trust (Central), Manchester, UK; ^8^Manchester Breast Centre, Manchester Cancer Research Centre, University of Manchester, Manchester, UK

**Correspondence:** D. G. Evans

**Aim**

To better stratify breast cancer risks to enable more targeted early detection/prevention strategies particularly to balance the risks/benefits of population.

**Method**

Data from 9,362 women unaffected by breast cancer at study entry who provided a DNA sample for polygenic-risk-score (PRS) were analysed from the 57,902 women in the PROCAS study. The PRS score was analysed along with mammographic density (density residual-DR) and standard risk factors to assess future risk of breast cancer pathological type and hormonal receptor status

**Results**

For the 195 prospective breast cancers a predictor based on Tyrer-Cuzick/DR/PRS was informative for subsequent cancer overall and more so for stage 2+ cancers and calibrated (0.99) for predicting cancers across all risk groups. Although DR was most predictive for HER2+ and stage 2+ cancers it did not discriminate as well between poor prognosis cancers and extremely good prognosis cancers as Tyrer-Cuzick or the PRS, with the PRS providing the highest OR for post-prevalent stage 2+ cancers IQR OR=1.79 (95%CI:1.30-2.46).

**Conclusion**

A combined approach using Tyrer-Cuzick, mammographic density and a PRS provides accurate risk stratification not only overall but also for worse prognosis cancers. This provides support for reducing screening intervals in the high and increasing them in the low risk groups.

## N5 Exogenous and endogenous associated factors to early onset colorectal cancer

### R. Zuppardo^1^, M. Di Leo^2^, A. Mannucci^1^, F. Azzolini^1^, D. Esposito^1^, L. Fanti^1^, G. Mazzoleni^1^, C. Notaristefano^1^, E. Viale^1^, R. Rosati^3^, P. Testoni^1^, G. M. Cavestro^1^

#### ^1^Gastroenterology and Gastrointestinal Endoscopy, Division of Experimental Oncology, Vita-Salute San Raffaele University, IRCCS Ospedale San Raffaele Scientific Institute, Milan, Italy; ^2^Humanitas Clinical and Research Center, Department of Biomedical Sciences, Humanitas University, Milan, Italy; ^3^Department of Gastrointestinal Surgery, San Raffaele Hospital, Vita-Salute San Raffaele University, Milan, Italy

**Correspondence:** R. Zuppardo

**Aim**

Early onset colorectal cancers (eoCRC < 50 years), is projected to increase by as much as 90% and 140%, respectively by 2030, and germline mutations appear to account for only about 20%. We investigate the role of exogenous and endogenous risk factors as associated factors in eoCRCs.

**Method**

Clinical, anamnestic and pathological data were retrieved on eoCRC patients from 06/2017 to 04/2018, and compared with a group of late onset CRC (loCRC) of the same period.

**Results**

We enrolled 33 eoCRCs and 48 loCRCs, mean age 40.7 +/- 7.3 and 66.1 +/- 9.8, respectively (p<0.001), prevalence of females (54.5% in eoCRCs and 52.1% in loCRCs). Diagnostic delay was higher in eoCRC group: 42.4% of eoCRCs diagnosed in the 6th months from symptoms onset versus 100% of loCRC patients (p<0.001). Lynch syndrome was more frequent in eoCRC (12%) than loCRC group (0%) p=0.02. A statistically significant difference was found in alcohol habit, 66.7% of no-drinker in eoCRCs and 41.7% of loCRCs (p=0.04), and a trend through significance for no-smokers in eoCRCs.

**Conclusion**

CRC should be considered earlier for differential diagnosis in young patients. We confirmed alcohol as cofactor in development of eoCRC and we underlined that familiar history should be collected to identify mutations carriers.

## N7 Cancer risks by age and gender and survival after cancer in Path_MSH6 carriers: a Prospective Lynch Syndrome Database (PLSD) report

### J. Sampson^1^, J. P. Plazzer^2^, M. Dominguez-Valentin^3, 4^, S. Nakken^4^, T. Seppälä^5^, C. Engel^6^, S. Aretz^6^, H. K. Schackert^6^, W. Schmiegel^6^, N. Rahner^6^, M. von Knebel Doeberitz^6^, M. Loffler^6^, E. Holinski-Feder^6,^ I. Bernstein^7^, L. Sunde^7^, M. Jenkins^8^, D. G. Evans^9^, J. Burn^9^, L. Bertario^10^, G. M. Cavestro^10^, A. Lindblom^11^, A. Della Valle^12^, R. H. Sijmons^13^, W. H. de Vos tot Nederveen Cappel^13^, L.Katz^14^, N. Gluck^14^, K. Heinimann^15^, C. A. Vaccaro^16,^ F. Lopez-Koestner^17^, F. Balaguer^18^, E. Hovig^4^, F. Macrae^19^, G. Möslein^6^, J. P. Mecklin^5^, G. Capella^18^, Members of the PLSD project^20^, Pål Møller^4, 21^

#### ^1^Cardiff University, Cardiff, UK; ^2^Australia & InSiGHT database; ^3^PLSD curator , ^4^Norwaycarriers: a Prospective Lynch Syndrome ^5^Finlandcarriers: a Prospective Lynch Syndrome ^6^Germany,^7^Denmark,^8^Australia,^9^UK,^10^Italy,^11^Sweden; ^12^Uruguaycarriers: a Prospective Lynch Syndrome ^13^The Netherlands; ^14^Israel; ^15^Switzerland; ^16^Argentina; ^17^Chile; ^18^Spain; ^19^Australia; ^20^Interational; ^21^PLSD PI

**Correspondence:** J. Sampson

**Aim**

To determine cancer risks by age and gender and cancer survival in carriers of path_MSH6 variants.

**Method**

An independent cohort of class 4 or 5 path_MSH6 carriers was used to validate findings reported previously by PLSD. Data for individuals in the previous and validation cohorts who carried class 4 or 5 variants listed in the InSIGHT variant database were then combined and analysed by age and gender, deriving more precise risk and survival estimates to inform management.

**Results**

The validation cohort (N=425) provided 2,367 prospective observation years and confirmed previously reported cumulative risks for any cancer: 14% vs 18% at fifty years and 48% vs 53% at 70 years. The combined series of 841 carriers of class 4/5 path_MSH6 variants provided 5,205 prospective observation years. Cumulative risks at 75 years in males/females were: any cancer 42%/60%; colorectum 18%/20%; endometrium NA/41%; ovary NA/11%; stomach, duodenum, bileduct, pancreas 8%/4%; ureter,_kidney 2%/6%; bladder 8%/1%; prostate 9%/NA; breast NA/14%; brain 2%/1%. Ten-year crude survival following cancer was: colon 100%; rectum 86% and endometrium 90%. See www.PLSD.eu to calculate risks for individual patients by age and gender.

**Conclusion**

MSH6-associated Lynch syndrome has distinct characteristics with a high risk of endometrial cancer compared to other organs.

## N09 Worldwide study of cancer risks for Lynch syndrome: International Mismatch Repair Consortium (IMRC)

### M. Jenkins^1^, J.C. Reece^1^, G. Lee^1^, R. Haile^2^, G. Moslein^3^, F. Macrae^4^, A. Win^1^

#### ^1^University of Melbourne, Melbourne, Australia; ^2^Cedars-Sinai Medical Center, California, USA; ^3^Helios St. Josefs-Hospital Bochum-Linden, Germany; ^4^Royal Melbourne Hospital, Melbourne, Australia

**Correspondence:** M. Jenkins

**Aim**

The International Mismatch Repair Consortium (IMRC) was established to determine cancer risks by geographic region.

**Method**

Pedigree data for 6,436 Lynch syndrome families from 22 countries were submitted by researchers/clinicians throughout the world to the analysis team at the University of Melbourne. We estimated the cumulative risks (penetrance) by geographic region. We used a modified segregation analysis and adjusted for any ascertainment of families.

**Results**

Preliminary analysis suggest that for MLH1 mutations, the risk of colorectal cancer to age 70 is highest for carriers in Australasia (68% males, 55% females) and North America (61% males, 48% females) and lowest for carriers in South America (12% males, 10% females) and East Asia (20% males, 14% females). For MSH2, the patterns were similar, except for South America which had a high estimated average risk (82% males, 75% females).

**Conclusion**

Collection of MMR family data from many international sites has progressed well despite the challenges faces by sites to establish databases for epidemiological research with varying resources. Preliminary results suggest that cancer risks for people with Lynch syndrome differ by geographic region which is consistent with environmental modifiers for the disease and might justify region specific screening guidelines.

## N10 Breast cancer risk in neurofibromatosis type 1 is a function of the type of NF1 gene mutation: a new genotype-phenotype correlation

### I. Frayling^1^, V. F. Mautner^2^, R. van Minkelen^3^, R. A. Kallionpää^4^, S. Aktaş^5^, D. Baralle^6^, S. Ben-Shachar^7^, A. Callaway^8^, H. Cox^1^, D. M. Eccles^6^, S. Ferkal^9^, H. LaDuca^10^, C. Lázaro^11^, M. T. Rogers^1^, A. J. Stuenkel^10^, P. Summerour^10^, A. Varan^12^, Y. S. Yap^13^, J. Peltonen^4^, D. G. Evans^14, 15^, P. Wolkenstein^16^, M. Upadhyaya^1^

#### ^1^Institute of Cancer & Genetics, Cardiff University, Cardiff, UK; ^2^Neurofibromatose–Ambulanz–Hamburg, Gebäude O 54 im UKE, Martinistraße 52, 20246 Hamburg, Germany; ^3^Klinische Genetica, Erasmus MC, Rotterdam, Netherlands; ^4^Department of Cell Biology and Anatomy, University of Turku, Turku, Finland; ^5^Dokuz Eylül Üniversitesi, Izmir, Turkey; ^6^Faculty of Medicine, Southampton University, Southampton, UK; ^7^The Genetic Institute, Tel-Aviv Sourasky Medical Center, Tel-Aviv, Israel; ^8^Wessex Regional Genetics Laboratory, Salisbury NHS Foundation Trust, Salisbury, UK; ^9^Service de Santé Publique, Hôpital Henri Mondor, Créteil, France ; ^10^Ambry Genetics, Aliso Viejo, California, USA; ^11^Institut Català d’Oncologia, L’Hospitalet, Spain; ^12^Hacettepe Üniversitesi Kanser Enstitüsü, Turkey; ^13^Department of Medical Oncology, National Cancer Centre, Singapore; ^14^Manchester Centre for Genomic Medicine, Central Manchester University Hospitals NHS Foundation Trust, Manchester, UK; ^15^Manchester Centre for Genomic Medicine, University of Manchester, Manchester, UK; ^16^Immunity Transplantation Infections, Hôpital Henri Mondor, APHP University Paris Est Créteil, Créteil, France

**Correspondence:** I. Frayling

**Aim**

NF1 predisposes to breast cancer (BC), but no genotype-phenotype correlations have been described.

**Method**

Constitutional NF1 mutations in 78 NF1 patients with BC (NF1-BC) were compared to the NF1 LOVD (N=3432).

**Results**

There are no gross relationships with mutation position. No cases were observed with large deletions (HR=0.10; 95%CI: 0.006–1.63; p=0.014, Fisher’s exact (FE)) 64.3% of the 70 different mutations have p<0.05 (FE), while 74.3% are significant when adjusted for multiple comparisons (Benjamini-Hochberg p≤0.125). Two pairs of patients shared the same predicted effects on neurofibromin, but had different mutations at the DNA level. 6/14 (43%) of the missenses (MS) were located in the CSRD (p=0.093; FE). 10/11 (91%) of MS cases with known age of BC occurred <50y (p=0.041; FE). 18 had BRCA1/2 testing, revealing one BRCA2 mutation.

**Conclusion**

This demonstrates that certain heritable mutation types, and indeed certain specific mutations in NF1 confer different risks of BC. The observation that NF1 amplification does not always occur with, and can occur independently of ERBB2 amplification, supports the concept that BC risk in NF1 may be due to gain of function mutations. A prospective NF1-BC study needs to be established. Regardless of NF1 mutation status NF1-BC patients warrant testing of other BC-predisposing genes.

## N11 A dominantly inherited 5’UTR variant causing methylation associated silencing of BRCA1 as a novel cause of breast and ovarian cancer

### D. G. Evans^1, 2, 3, 4, 8^, E. M. van Veen^1, 5, 8^, H. J. Byers^1, 5^, A. J. Wallace^5^, J. M. Ellingford^1, 5^, G. Beaman^1, 5^, J. Santoyo-Lopez^6^, T. J. Aitman^6^, D. M. Eccles^7^, F. I. Lalloo^5^, M. J. Smith^1, 5, 8^, W. G. Newman^1, 4, 5, 8^

#### ^1^Division of Evolution and Genomic Sciences, School of Biological Sciences, Faculty of Biology, Medicine and Health, University of Manchester, Manchester Academic Health Science Centre, Manchester, UK; ^2^Prevention Breast Cancer Centre and Nightingale Breast Screening Centre, University Hospital of South Manchester, Manchester, UK; ^3^The Christie NHS Foundation Trust, Manchester, UK; ^4^Manchester Breast Centre, Manchester Cancer Research Centre, University of Manchester, Manchester, UK; ^5^Manchester Centre for Genomic Medicine, St. Mary’s Hospital, Manchester University NHS Foundation Trust, Manchester Academic Health Science Centre, Manchester, UK; ^6^Centre for Genomic and Experimental Medicine, and Edinburgh Genomics, University of Edinburgh, Edinburgh, UK; ^7^Cancer Sciences Academic Unit and Southampton Clinical Trials Unit, Faculty of Medicine, University of Southampton and University Hospital Southampton Foundation Trust, Southampton, UK; ^8^Department of Genomic Medicine, St Mary's Hospital, Manchester Universities NHS Foundation Trust Manchester, UK

**Correspondence:** D. G. Evans

**Aim**

Pathogenic variants in BRCA1/BRCA2 are identified in ~20% of families with multiple individuals with early-onset breast/ovarian cancer. Extensive searches for additional highly penetrant genes/alternative mutational mechanisms altering BRCA1/2 have not explained the missing heritability. For the first time, we report a dominantly inherited 5’UTR variant associated with epigenetic silencing of BRCA1 due to promoter hypermethylation in two families with breast/ovarian cancer.

**Method**

BRCA1 promoter methylation of ten CpG dinucleotides in breast/ovarian cancer families without germline BRCA1/2 pathogenic variants was assessed by pyrosequencing and clonal bisulfite sequencing. BRCA1 RNA/DNA sequencing from lymphocytes was undertaken to establish allelic expression and germline variants.

**Results**

BRCA1 promoter hypermethylation was identified in 2/49 families with multiple women affected with grade-3 breast/high-grade-serous ovarian cancer. Soma-wide BRCA1 promoter hypermethylation was confirmed in blood/buccal mucosa/hair follicles. Methylation levels were ~50%, consistent with complete silencing of one allele. RNA sequencing revealed allelic BRCA1 expression loss in both families segregating with a novel heterozygous variant c.-107A>T in 5’UTR.

**Conclusion**

Our results indicate a novel mechanism for familial breast/ovarian cancer, caused by an in cis 5’UTR variant associated with epigenetic silencing of BRCA1 promoter. We propose methylation analyses are undertaken to establish the frequency of this mechanism in families affected by early onset breast/ovarian cancer without a BRCA1/2 pathogenic variant.

## N13 Identification Of genetic variants in early-onset and familial cancers by targeted next generation sequencing

### M. Dominguez-Valentin^1^, S. Nakken^1^, H. Tubeuf^2^, D. Vodak^1^, P. O. Ekstrøm^1^, A. M. Nissen^3, 4^, M. Morak^3, 4^, E. Holinski-Feder^3, 4^, A. Holth^5^, B. Davidson^5, 6^, A. Martins^2^, P. Møller^7, 8^, E. Hovig^1^

#### ^1^Department of Tumor Biology, Institute for Cancer Research, Oslo University Hospital, Oslo, Norway; ^2^Inserm-U1245, UNIROUEN, Normandie Univ, Normandy Centre for Genomic and Personalized Medicine, Rouen, France; ^3^Medizinische Klinik und Poliklinik IV, Campus Innenstadt, Klinikum der Universität München, Ziemssenstr. 1, Munich, Germany; ^4^MGZ—Medizinisch Genetisches Zentrum, Munich, Germany; ^5^Department of Pathology, Oslo University Hospital, Norwegian Radium Hospital, Oslo, Norway; ^6^University of Oslo, Faculty of Medicine, Institute of Clinical Medicine, N-0316 Oslo, Norway; ^7^Department of Human Medicine, Universität Witten/Herdecke, Germany; ^8^Department of Medical Genetics and Tumor Biology, Oslo University Hospital, Norway

**Correspondence:** M. Dominguez-Valentin

**Aim**

To study the potential contribution of genes other than BRCA1/2, PTEN, TP53 and MMR to the biological and clinical characteristics of early-onset and familial cancers in Norwegian families.

**Method**

The Hereditary Cancer Biobank from the Norwegian Radium Hospital was used to identify early-onset families and individuals with a high risk of developing breast, gynecological and colorectal cancers. Forty-four cancer susceptibility genes were selected and analyzed by our in-house designed TruSeq amplicon-based assay for targeted sequencing. Protein- and RNA splicing-dedicated in silico analyses were performed for all variants of unknown significance (VUS). Variants predicted as the more likely to affect splicing were experimentally analyzed by a minigene assay (PMID: 29458332, 29371908, 28608266).

**Results**

We analyzed 176 early onset and familial cases who harbored 5% (8/175) class 5 variants in the genes ATM (3), CHEK2 (2), MSH6, MUTYH and MAP3K1. Out of the 18 VUS tested in the minigene splicing assay, ATM c.3806A>G, NOTCH3 c.14090C>T and MSH2 c.815C>T showed a significant effect on RNA splicing.

**Conclusion**

Our study provides new information on genetic loci that may affect the risk of developing cancer in these patients and their families, demonstrating that genes presently not routinely tested in molecular diagnostic settings may be important for capturing cancer predisposition in these families.

## N14 Identification and characterization of an alu element insertion in BRCA2 in a Spanish family associated to prostate cancer

### V. Barca-Tierno^1^, M. Morín^1^, L. Santos^1^, C García-Hoz^3^, L. Fuente-García^1^, P. Marcos-Cava^1^, M. Salazar^2^, C. Guillén-Ponce^2^, M. A. Moreno-Pelayo^1^

#### ^1^Genetics Service, Ramon and Cajal Hospital-IRYCIS-CIBERER Raregenomics; ^2^Oncology Service, Ramon and Cajal Hospital; ^3^Immunology Service, Ramon and Cajal Hospital

**Correspondence:** V. Barca-Tierno

**Aim**

Pathogenic Alu element insertions are rarely reported because this type of insertions are undetectable with the classical screening methods. The aim of this work has been the identification and characterization of an Alu element insertion in a Spanish family with a history of breast/ovarian cancer.

**Method**

Molecular analysis was carried out using the BRCA MASTRDx (Multiplicom) and Massively Parallel Sequencing (Illumina). The Alu insertion was identified and characterized by fragments analysis, genotyping, PCR amplification and Sanger sequencing (ABI3130).

**Results**

We have identified and characterized a heterozygous pathogenic variant c.5007_5008ins174 located at the exon 11 of the BRCA2 gene in a patient with prostate cancer. The variant identified is a pathogenic Alu element insertion (AluYb8BRCA2) of about 174 bp long.

**Conclusion**

NGS has been incorporated into clinical genetic testing for hereditary cancer risk. NGS-based techniques and the standard bioinformatic pipelines, however, are unable to detect and precissely characterize ALU element insertions. In this work, we report, by using classical screening methods and bioinformatic programs, BLAST and RepeatMasked, the identification of the AluYb8BRCA2 insertion in BRCA2 coding region. This insertion could generate a framesift resulting in the abrogation of BRCA2 protein function that has been associated with oxidative stress involved in carcinogenesis.

## N18 Deciphering the contribution of recently proposed polyposis predisposing genes

### M. Terradas^1, 2^, P. M. Muñoz^1, 2^, S. Belhadj^1, 2^, G. Aiza^1, 2, 3^, M. Navarro^1, 2, 3^, S. González^1, 2, 3^, E. Darder^4^, J. Brunet^1, 3, 4^, M. Pineda^1, 2,3^, G. Capellá^1, 2, 3^, L. Valle^1, 2, 3^

#### ^1^Hereditary Cancer Program, Catalan Institute of Oncology, IDIBELL, 08908 Hospitalet de Llobregat, Barcelona, Spain; ^2^Program in Molecular Mechanisms and Experimental Therapy in Oncology (Oncobell), IDIBELL, 08908 Hospitalet de Llobregat, Barcelona, Spain; ^3^Centro de Investigación Biomédica en Red de Cáncer (CIBERONC), Spain; ^4^Hereditary Cancer Program, Catalan Institute of Oncology, IDIBGi, 17007 Girona, Spain

**Correspondence:** M. Terradas

**Aim**

The genetic defect responsible for colorectal polyposis remains unknown in much of the cases with adenomatous polyposis. Recently, MCM9 (recessive), FOCAD (recessive or dominant) and POLQ (dominant) have been identified as putatively new polyposis genes. Here we aim at providing a more definitive answer about the contribution of germline mutations in these genes to adenomatous polyposis.

**Method**

A total of 182 unrelated polyposis patients were screened for MCM9, FOCAD and POLQ mutations using PCR amplification in pooled DNAs combined with targeted parallel sequencing. Variants detected in the pooled samples were validated by genotyping and/or Sanger sequencing.

**Results**

While no homozygotes or compound heterozygotes where identified in MCM9 and FOCAD, a predicted deleterious missense variant (c.911A>G; p.N304S) was identified in heterozygosis in MCM9 in an individual with adenomatous polyposis, and 4 were identified in the FOCAD gene: c.401C>T (p.P134L), c.1393G>A (p.G465R), c.2861C>T (p.P954L) and c.3041A>G (p.Y1014C). A stop-gain variant (c.7537C>T; p.Q2513*) located in the DNA-polymerase domain and a predicted deleterious missense variant (c.4684G>T; p.D1562Y), were identified in POLQ.

**Conclusion**

Additional studies are currently being performed in order to elucidate the association of the identified variants with the predisposition to polyposis in the carrier families.

## N19 Colorectal cancer risk is not increased in NTHL1 heterozygous mutation carriers

### A. Ragunathan^1, 3^, M. Clendenning^1^, K. Mahmood^1^, B. J. Pope^1^, D. J. Park^1^, H. Jayasekara^1^, J. E. Joo^1^, C. Rosty^2^, T. Green^1^, S. Preston^1^, N. O’Callaghan^1^, F. A. Macrae^3^, I. M. Winship^3^, A. K. Win^1^, J. L. Hopper^1^, P. Newcomb^4^, S. Gallinger^5^, M. A. Jenkins^1^, D. D. Buchanan^1^

#### ^1^University of Melbourne, Melbourne, Australia; ^2^University of Queensland, Queensland, Australia; ^3^The Royal Melbourne Hospital, Melbourne, Australia; ^4^Fred Hutchinson Cancer Research Center, Washington, USA; ^5^Lunenfeld Tanenbaum Research Institute, Mount Sinai Hospital, New York, USA

**Correspondence:** A. Ragunathan

**Aim**

Bilallelic loss-of-function germline mutations in the base excision repair gene NTHL1 result in an increased risk of colorectal polyps and different cancer types, resulting in the inclusion of this gene on many multi-gene cancer predisposition panels. However, the impact of heterozygous germline NTHL1 mutations on colorectal cancer (CRC) risk is unclear.

**Method**

1953 CRC-affected individuals and 1207 controls from the Colon Cancer Family Registry Cohort were screened for coding single nucleotide and short indels variants in NTHL1 using a targeted multiplex PCR-based sequencing approach (Hi-PLEX). Variants were filtered on sequencing depth and allele proportions. Variants were predicted to be pathogenic if they were novel or rare (gnomAD < 0.05%), protein truncating variants or missense variants predicted to be deleterious (based on CADD>20 or REVEL>0.5).

**Results**

We detected 22 (1.13%) predicted pathogenic variants in cases and 17 (1.41%) in controls (OR=0.79, 95% CI=0.42-1.48, p=0.51), all carriers were heterozygotes. The loss-of-function variants identified were not different in frequency between CRC cases (n=5, 0.26%) and controls (n=5, 0.41%; OR=0.62, 95%CI=0.18-2.14, p=0.52), and of similar frequency to rare NTHL1 loss-of-function variants observed in gnomAD (0.199%).

**Conclusion**

The effect of heterozygous NTHL1 predicted pathogenic variants on CRC risk, if any, is not likely to be more than 1.5 fold.

## N22 Family case of rare MSH6 variant identified as secondary finding - shall we screen for Lynch?

### Tomas Szemes^1, 2, 3^

#### ^1^Geneton Ltd., Bratislava, Slovak Republic; ^2^Comenius University Science Park, Bratislava, Slovak Republic; ^3^Faculty of Natural Sciences, Bratislava, Slovak Republic

**Aim**

In Slovakia, the incidence of colorectal cancer is one of the highest worldwide and could be a result of higher incidence of cancer predisposing syndromes, such as Lynch syndrome. Novel large gene panel, exome or whole genome tests become less costly and widely available which allow detection of cancer predisposing genetic variants. In addition, novel non-invasive methods for tumor screening (liquid biopsy) become available as well.

Screening for genetic cancer predisposing syndromes and accordingly adjusted cancer screening regimes with inclusion of liquid biopsy methods could be viable options but the implementation and social aspects need to be studied.

**Method**

A clinical exome test was carried out in 20yo male. Identification of variants relevant for secondary findings was carried out too. Identified variants were verified by Sanger sequencing. A family follow-up included clinical exome test as well as Sanger confirming tests. Written informed consent to publish was obtained from the patients involved in this study.

**Results**

We identified a rare potentially pathogenic variant in MSH6 gene in 20yo male as secondary finding. In addition a rare BRCA2 variant was also detected and confirmed. Despite family cancer history did not meet used criteria it was tested for these variants.

A suspected case of metastatic breast cancer in the family was confirmed in 65yo female bearing the in 12/2017 and a suspected case od CRC was identified in 67yo male bearing the MSH6 variant.

All family members adherred to required medical procedures after genetic testing.

**Conclusion**

Genomic tests and their wider availability with novel liquid biopsy methods offer novel cancer screening algorithm options. A case of family with two detected variants in both BRCA2 and MSH6 a secondary finding shows possible benefits but social aspects have to be considered for wider implementation.

## N24 Systematic linkage of all diagnostic hereditary cancer genotypes to the National Cancer Registry

### F. McRonald^1^, B. Shand^1, 2^, N Bricault^2^, S. Vernon^1^, J. Rashbass^1, 2^, J. Burn^3^

#### ^1^Public Health England, UK; ^2^Health Data Insight CiC, Cambridge, UK; ^3^Northern Genetics Service, Newcastle, UK

**Correspondence:** F. McRonald

**Aim**

To create a national service collecting pseudonymised germline cancer-predisposing genotypes, and link these to the National Cancer Registration and Analysis Service for individuals with a prior or subsequent cancer diagnosis.

**Method**

NHS molecular genetics laboratories submit patient-level genotype data through a secure online portal. Unique patient demographics are pseudonymised using a one-way hash function that generates an irreversible pseudoID; additional identifiers are secondarily encrypted. The same hash function applied to cancer registration records, where patient identity is already known, enables linkage of the genotype data; decryption of additional identifiers is then possible. We can thus obtain accurate variant counts nationally, and identify those who develop cancer, without compromising patient privacy.

**Results**

Pilot work has focused upon BRCA1 and BRCA2 genes; we are now commencing collection of colorectal cancer predisposition gene data. To date, ten laboratories have submitted BRCA1/2 data, covering a time period from 2001 onwards, and including ~1300 different gene variants. Initial linkage to cancer registry records showed a 68% match rate.

**Conclusion**

This robust, secure system collects depersonalised but linkable genotypes on all individuals tested. Record-level linkage to the rich phenotype, treatment and outcome data in the national cancer registry provides allelic frequency and associated phenotype data, and facilitates variant interpretation.

## N27 The management of gynaecological cancers in Lynch syndrome: the Manchester International Consensus Meeting

### E. J. Crosbie, N. A. J. Crosbie, D. G. Evans

#### University of Manchester, Manchester, UK

**Correspondence:** E. J. Crosbie

**Aim**

There are no internationally agreed clinical guidelines as to how best to manage the risk, prevention and treatment of gynaecological cancers in women with Lynch syndrome. The Manchester International Consensus Group was convened in April 2017 to develop clear and comprehensive clinical guidance regarding the management of the gynaecological sequelae of Lynch syndrome based on existing evidence and expert opinion from medical professionals and patients.

**Method**

Stakeholders from Europe and North America worked together over a two-day workshop to achieve consensus on best practice. Stakeholders included patients, patient support groups, gynaecologists, clinical geneticists, medical oncologists, colorectal surgeons, gastroenterologists, histopathologists, genetic pathologists, health economists, epidemiologists, gynaecology nurse specialists and genetic counsellors.

**Results**

Guidance was developed in four key areas: 1) whether women with gynaecological cancer should be screened for Lynch syndrome and 2) how this should be done; 3) whether gynaecological surveillance was of value for women with Lynch syndrome; and 4) what preventive measures should be recommended for women with Lynch syndrome to reduce their gynaecological cancer risk.

**Conclusion**

The Manchester International Consensus Guideline provides comprehensive clinical guidance that can be referenced by both patients and clinicians so that women with Lynch syndrome can expect and receive appropriate standards of care.

**Acknowledgements**

Manchester International Consensus Group

## N28 Awareness of Lynch like syndrome within clinical genetics – results from a UK survey

### D. Georgiou^1^, V. Kiesel^1^, A. F. Brady^1^, K. Monahan^2^

#### ^1^North West Thames Regional Genetics Service, UK; ^2^Chelsea and Westminster Hospitals NHS Foundation Trust, UK

**Correspondence:** D. Georgiou

**Aim**

UK NICE guidelines (2017) recommend screening of all new colorectal cancers with either immunohistochemistry (IHC) or Microsatellite instability (MSI) testing. Following an abnormal IHC or MSI result, up to 70% of individuals may have no identifiable germline mutation. This group constitutes Lynch-like syndrome (LLS), estimated to represent 3% of colorectal cancer cases.

Evidence suggests the majority of LLS cases are caused by somatic variants in the tumour. Colorectal and extracolonic cancer risks in LLS are increased in comparison to population risks. UK guidelines suggest 2 yearly colonoscopies for individuals with LLS and their first degree relatives; assuming there may be an unknown hereditary cause.

**Method**

We conducted a survey amongst clinicians practising in regional clinical genetics departments within the UK. We aimed to explore clinicians’ understanding and management of LLS families. The survey was disseminated by the cancer lead clinician within each department, through a “surveymonkey” link.

**Results**

We received 44 responses from 19 centres. 40% of participants were aware of the definition of LLS while 27% would offer 2 yearly colonoscopies. There were variations in practice within and between departments.

**Conclusion**

These results emphasize the importance of increasing awareness of LLS, and contribute towards the need for clear management guidelines.

## N30 Life-long immune surveillance and immunoediting – evidence From Lynch syndrome cancers

### M. Kloor^1^, M. Ozcan^2^, J. Janikovits^2^, F. Echterdiek^2^, A. Ahadova^2^, J. Krzykalla^3^, A. Benner^3^, M. von Knebel Doeberitz^2^

#### ^1^Department of Applied Tumor Biology, University Hospital Heidelberg; ^2^University Hospital Heidelberg; ^3^German Cancer Research Center

**Correspondence:** M. Kloor

**Aim**

Lynch syndrome-associated cancers accumulate a high load of immunogenic frameshift peptide neoantigens as a consequence of DNA mismatch repair (MMR) deficiency. MMR-deficient cells can therefore be recognized by the immune system. We aimed to comprehensively characterize the immune phenotype of MSI cancers, accounting for somatic mutations inducing immune evasion and for immune cell infiltration.

**Method**

We combined the analysis of our own cohort of MSI cancers with mutation data of MSI cancers of the TCGA/DFCI cancer collections. Immune cell infiltration was quantified by immunohistochemistry, using antibodies specific for T cell subtypes, including CD3, FOXP3, and PD-1.

**Results**

72% of MMR-deficient colorectal cancers of the DFCI database harbored alterations affecting genes involved in HLA class I-mediated antigen presentation. The most common alterations were truncating mutations affecting the Beta2-microglobulin (B2M) gene. B2M mutations were related to a higher density of activated T cells infiltrating the tumor, and to a lower frequency of regulatory T cells in the tumor environment.

**Conclusion**

The extraordinarily high prevalence of immune evasion phenomena in MSI cancer most likely reflects life-long immune surveillance and suggests that most MSI pre-cancers are eliminated by the immune system if they fail to evade the immune attack.

## N32 A novel mainstreaming model for Lynch syndrome genetic testing in colorectal cancer patients

### D. Georgiou^1^, B. Desouza^2^, A. F. Brady^1^, N. Ellery^1^, C. Berlin^1^, A. Latchford^3^, S. Clark^3^, H. J. Thomas^4^

#### ^1^North West Thames Regional Genetics Service; ^2^South East Thames Regional Genetics Service; ^3^Polyposis Registry, St Marks Hospital; ^4^Family Cancer Clinic, St Marks Hospital

**Correspondence:** D. Georgiou

**Aim**

New NICE guidance (2017) recommends universal tumour screening for Lynch syndrome (LS) in all patients with newly diagnosed colorectal cancer (CRC). Identifying CRCs with deficient DNA mismatch repair (dMMR) will guide further diagnostic testing for LS. Establishing a diagnosis of LS has important implications for the management of CRC patients. All CRC patients with suspected LS should have access to appropriate diagnostic testing performed within a suitable time frame. Based on the anticipated rapid increase in clinical need, we have developed and implemented a novel mainstreaming model for LS genetic testing.

**Method**

Reflex IHC testing is undertaken in all newly diagnosed CRC cases; abnormal results are reviewed at the colorectal MDM, and eligible patients offered germline testing at their routine cancer appointments by appropriately trained cancer clinicians (i.e. oncologists and surgeons). Genetic results are fed back to patients by the cancer team, and all patients with a pathogenic variant or a variant of unknown significance are referred to clinical genetics for further management.

**Results**

We present the pathway as adopted at St Marks Hospital and the outcomes from the first year post implementation.

**Conclusion**

This pathway was effective at our hospital.

## N33 Validation and updating of Path_MLH1 in cases with class 4 And 5 genetic variants; a Prospective Lynch Syndrome Database (PLSD) Report

### T. Seppälä^1^, J. P. Plazzer^2^, M. Dominguez-Valentin^3^, S. Nakken^4^, C. Engel^5^, S. Aretz^5^, M. A. Jenkins^6^, L. Sunde^7^, I. Bernstein^7^, F. Balaguer^8^, A. Lindblom^9^, D. G. Evans^10,^ L. Bertario^11^, J. Burn^12^, E. Holinski-Feder^13^, F. Lopez-Koestner^14^, A. Della Valle^15^, K. Heinimann^16^, C. A. Vaccaro^17^, W. H. de Vos tot Nederveen Cappel^18^, R. H. Sijmons^19^, N. Gluck^20^, L. Katz^20^, G. M. Cavestro^11^, E. Hovig^4^, F. Macrae^21^, G. Möslein^22^, J. Sampson^23^, G. Capella^8^, J. P. Mecklin^1^, P. Møller^24^

#### ^1^Finland; ^2^InSiGHT database, Australia; ^3^PLSD curator Norway; ^4^Norway; ^5^German HNPCC consortium; ^6^Colon Cancer Family Registry: Australia, USA, Canada; ^7^Denmark; ^8^Spain; ^9^Sweden; ^10^Manchester; United Kingdom; ^11^Italy; ^12^Newcastle, United Kingdom; ^13^Münich, Germany; ^14^Chile; ^15^Uruguay; ^16^Switzerland; ^17^Argentina; ^18^Leiden; Holland; ^19^Groningen, Holland; ^20^Israel; ^21^Melbourne, Australia; ^22^Germany; ^23^Cardiff, United Kingdom; ^24^PLSD PI; OUS Norway, Witten-Herdecke, Germany

**Correspondence:** T. Seppälä

**Aim**

Determine average risks for and survival after cancer in path_MLH1 carriers.

**Method**

Previously reported results were validated in an independent series of path_MLH1 carriers followed-up by colonoscopy. Combined results merging former and present series included only carriers with pathogenic class 4 or 5 variants listed in the InSiGHT database.

**Results**

The validation series including 10,037 observation years confirmed previously published cumulative risk for any cancer: at fifty years, 37% in the validation series compared to 40% in the previous series, and at 75 years, 78% compared to 76%. The combined series of path_MLH1 variant carriers included 24,297 observation years. Cumulative risk for cancer in specific organs or group of organs at 75 years were (males/females): Any cancer 70%/80%; colon_rectum 56%/47%; endometrium -/37%; ovaries -/11%; stomach_duodenum_bileduct_pancreas 21%/11%; ureter_kidney 4.7%/3.6%; urinary bladder 6.4%/4.9%; prostate 12%/-; breast -/12%; brain 0.7%/1.6%. Ten-year crude survival after cancer in different organs were: colon 86%; endometrium 90%; ovaries 82%; ureter_kidney 61%; urinary bladder 54%; prostate 90%, breast 80%; brain 0%.

**Conclusion**

PLSD reports average risks for and survival after cancer in path_MLH1 carriers of variants classified as clinically actionable in the InSiGHT database. See www.PLSD.eu for risk determination in any single patient by age and gender.

## N35 An assesment of endometrial cancer risk markers in Lynch syndrome patients

### A. Alonso^1^, R. Guarch^1^, S. Moreno^1^, E. Recari^1^, M. Miranda^1^, I. Chirivella^2^, E. Lastra^3^, L. Robles^4^

#### ^1^Complejo Hospitalario Navarra, Spain; ^2^Hospital Clínico Universitario de Valencia, Spain; ^3^Hospital Universitario de Burgos, Spain; ^4^Universitario 12 Octubre Madrid, Spain

**Correspondence:** A. Alonso

**Aim**

Assesment of a set of markers to anticipate endometrial cancer occurence in healthy female Lynch syndrome (LS) carriers.

**Method**

Materials: 242 biopsy specimens obtained during the prospective annual follow-up of 79 Lynch syndrome (LS) carriers from 4 different Spanish Centers.

Investigated markers were (High Microsatellite Instability MSI-H; abnormal mismatch repair proteins (MMR-IHC) or PTEN (PTEN-IHC) inmunohistochemistry; LINE sequences (LINE-CIN) or MMR genes CpG islands abnormal methylation (MMR-MMR), and somatic mutations in a custom panel of 27 genes related to type 1 endometrial carcinogenesis (Panel-27).

**Results**

Simultaneous presence of abnormal MMR and PTEN-IHC anticipated the occurrence of the precursor lesion “focal hyperplasia” in a median time of 19,63 months (CI95=17,55-21,71) with a Hazard Ratio HR= 3,97 (CI95=1,32-11,9) vs the no markers group. Panel-27 somatic mutations rate was also higher (75x10-6 mutations per Mb vs. 12x10-6 mutation per Mb, p<0,05) in these samples.

**Conclusion**

These findings provide a basis for recommending to introduce the investigation of these markers in biopsy specimens from LS patients, as a supportive tool for selecting the most appropriate management option in these patients (prophylactic hysterectomy vs surveillance)

## N36 Back to back comparison of colonoscopy with virtual chromoendoscopy using third generation narrow band imaging system to chromoendoscopy with indigo carmine in Lynch syndrome patients

### E. Samaha^1^, C. Colas^2^, M. Dhooge^3^, J. C. Saurin^4^, T. Lecomte^5^, E. Coron^6^, G. Rahmi^1^, G. Perrod^1^, C. Savale^1^, S. Chaussade^3^, J. Bellanger^2^, N. Benech^4^, J. P. Barbieux^5^, M. Le Rhun^6^, H. Pereira^1^, G. Chatellier^1^, C. Cellier^1^

#### ^1^European Georges Pompidou Hospital; ^2^Saint Antoine Hospital; ^3^Cochin Hospital; ^4^Edouard Heriot Hospital; ^5^CHU Tours; ^6^CHU Nantes

**Correspondence:** E. Samaha

**Aim**

Colonoscopic screening with indigo carmine chromoendoscopy (ICC) in Lynch Syndrome (LS) patients improves adenoma detection rate and is widely used nowadays. Nevertheless, it is a time- and-money-consuming technique which requires a dedicated training. Narrow band imaging (NBI) is a well-known virtual chromoendoscopy technique that highlights superficial mucosal vessels and improves contrast for adenomas. We conducted a prospective multicenter study in a back-to-back fashion to compare 3rd generation NBI to ICC for detecting colonic adenomas in LS patients.

**Method**

One hundred and thirty eight patients underwent a double colonoscopy, first with NBI, followed by ICC, in a back-to-back fashion. All polyps detected in either pass were removed for histopathological analysis. The primary outcome measure was the number of patients with at least one adenoma after NBI compared with the number of patients with at least one adenoma after NBI and ICC. Proportions were compared with the paired exact test (McNemar’s test). Continuous variables were compared with the Wilcoxon signed-rank test.

**Results**

All of the 138 patients were proven MMR mutation carriers (MLH1 = 33%, MSH2 = 47%, MSH6 = 15%, PMS2 = 4%, EPCAM = 1%). Mean age (standard deviation [SD]) was 40.5 (14.7) years, 64 (46.4%) were male. The median time for an NBI procedure was 8 minutes (interquartile range [IQR] 6–11) compared to 13 minutes (IQR 8–17) for ICC. At least one adenoma was detected during the initial NBI pass in 28 (20.3%) of 138 patients. ICC detected additional adenomas in 25 (18.1%) of 138 patients. Forty-two patients (30.4%) had at least one adenoma detected after both NBI and ICC; this represents an increase of 50.0% of the adenoma detection rate (ADR) (p=0.0001). The total number of adenomas increased from 39 after NBI pass to 75 after ICC pass with a mean number of adenomas detected per patient of 0.3 (0.7) after NBI pass vs 0.5 (1.1) after both NBI and ICC passes (p<0.0001). The ADR for flat adenomas was 10.9% after NBI vs 23.2% after ICC (p<0.0001). The ADR increased for right sides adenomas (10.9% after NBI vs 16.7% after ICC, p=0.0078) as well as for diminutive adenomas ≤5mm (16.7% after NBI vs 28.3% after ICC, p<0.0001). Detection of both sessile adenomas (11.6% NBI vs 13.8% ICC, p=0.25) and adenomas > 5mm (6.5% NBI vs 8.0% ICC, p=0.5) did not differ significantly between the 2 techniques. After adding white light detected adenomas, the total ADR of the study was 33.3%.

**Conclusion**

Colonoscopy with indigo carmine chromoendoscopy detects significantly more adenomas than 3rd generation NBI in LS patients, whereas sessile and > 5mm adenomas are equally detected. Although less time consuming, NBI colonoscopy cannot be recommended to replace indigo carmine chromoendoscopy in LS patients.

## N37 Cancer incidences by age in Path_PMS2 carriers: a report from the Prospective Lynch Syndrome Database (PLSD)

### M. Dominguez-Valentin^1^, S. W. Ten Broeke^2^, J. P. Plazzer^3^, T. Seppälä^4^, S. Nakken^1^, M. A. Jenkins^5^, R. H. Sijmons^6^, G. Capella^7^, C. Engel^8^, S. Aretz^9^, L. Sunde^10^, I. Bernstein^11^, D. G. Evans^12^, J. Burn^13^, M. Greenblatt^14^, F. Balaguer^15^, M. Grazia Tibiletti^16^, E. Holinski-Feder^17^, H. K. Schackert^18^, W. Schmiegel^19^, N. Rahner^20^, M. Löffler^8^, F. Macrae^3^, J. Sampson^21^, H. Thomas^22^, A. Lindblom^23^, W. H. de Vos tot Nederveen Cappel^24^, F. Lopez-Koestner^25^, A. Della Valle^26^, E. Hovig^1^, G. Möslein^27^, J. P. Mecklin^28^, M. Nielsen^2^, P. Møller^1, 29, 30^

#### ^1^Oslo University Hospital, Oslo, Norway; ^2^Leiden University Medical Center, The Netherlands; ^3^Melbourne University, Melbourne, Australia; ^4^University of Helsinki, Helsinki, Finland; ^5^Colon Cancer Family Registry, Australia; ^6^University Medical Center Groningen, Groningen, The Netherlands; ^7^L'Hospitalet de Llobregat, Barcelona, Spain; ^8^University of Leipzig, Leipzig, Germany; ^9^University of Bonn, Bonn, Germany; ^10^Aarhus University Hospital, Denmark; ^11^Aalborg University Hospital, Aalborg, Denmark; ^12^University of Manchester, London, UK; ^13^Newcastle University, Newcastle upon Tyne, UK; ^14^University of Vermont College of Medicine, USA; ^15^Universitat de Barcelona, Barcelona, Spain; ^16^Università dell'Insubria, Varese, Italy; ^17^Center of Medical Genetics, Munich, Germany; ^18^University of Dresden, Germany; ^19^University of Bochum, Germany; ^20^University of Düsseldorf, Germany; ^21^Cardiff University School of Medicine, Heath Park, UK; ^22^Imperial College London, London, UK; ^23^Karolinska Institutet, Stockholm, Sweden; ^24^Isala Clinics, Zwolle, The Netherlands; ^25^Clinica Las Condes, Chile; ^26^Hospital de las Fuerzas Armadas, Uruguay; ^27^University Witten-Herdecke, Wuppertal, Germany; ^28^University of Jyväskylä, Jyväskylä, Finland; ^29^PLSD PI, ^30^Witten-Herdecke, Germany

**Correspondence:** M. Dominguez-Valentin

**Aim**

Determine average risks for cancer in path_PMS2 carriers.

**Method**

Prospectively observed cancers in carriers of PMS2 variants classified as pathogenic (class 4/5) in the InSiGHT database.

**Results**

407 carriers were prospectively observed for 2239 years and they underwent regular surveillance and if needed polypectomies. Cumulative incidences for cancer at 50/75 years of age were: Any cancer 8% (95% CI 0%-19%)/32% (95% CI 14%-50%); colorectal- 0% /9% (95% CI 0%-21%); endometrial 0%/13% (95% CI 1%-25%); ovarian 0%/3%(95% CI 0%-9%); and urinary tract 0%/3% (95% CI 0%-9%) cancer.

**Conclusion**

Neither colorectal, endometrial, ovarian nor urinary tract cancer was observed before 50 years of age. The point estimates for colorectal and endometrial cancers at age 75 were, however, higher than expected despite undergoing regular surveillance . The patients examined were mostly selected from cancer kindreds, and the late onset cancers might not necessarily have been caused by the path_PMS2 variants. Clinical guidelines for monoallelic path_PMS2 carriers should be revised.

## N38 Yield of Lynch syndrome surveillance for individual MMR genes

### A. Goverde^1^, A. Wagner^1^, E. Viskil^1^, M. J. Bruno^2^, R. M. W. Hofstra^1^, M. C. W. Spaander^2^

#### ^1^Department of Clinical Genetics. Erasmus MC, University Medical Center, Rotterdam, The Netherlands; ^2^Department of Gastroenterology and Hepatology, Erasmus MC, University Medical Center, Rotterdam, The Netherlands

**Correspondence:** A. Wagner

**Aim**

To assess the yield of Lynch syndrome (LS) surveillance for MLH1, MSH2, MSH6 and PMS2 mutation carriers.

**Method**

Data on colonoscopy surveillance was collected for all LS mutation carriers in our center. We compared the development of adenomas and CRC between the different gene mutation carrier groups.

**Results**

Colonoscopy data was available for 264/314 (84%) patients; 55 MLH1, 44 MSH2, 143 MSH6 and 22 PMS2 mutation carriers. Median age was 44 years (IQR 35-56 years) and median follow-up time 6 years (IQR 2-10 years). At first colonoscopy CRC was found in eight patients and during 916 follow-up colonoscopies in nine patients. No CRC was found in MSH6 or PMS2 mutation carriers. There were no significant differences in the number of colonoscopies with adenomas or advanced adenomas between the different gene mutation carrier groups. In MSH6 mutation carriers advanced neoplasia (advanced adenoma or colorectal carcinoma) was found after a longer follow-up time than in the other mutation carrier groups.

**Conclusion**

Since no CRC was found during follow-up in MSH6 mutation carriers and advanced neoplasia was found in shorter follow-up time in MLH1 and MSH2 mutation carriers, the colonoscopy interval in MSH6 mutation carriers might be less stringent than for MLH1 and MSH2 mutation carriers.

## N39 The Prospective Lynch Syndrome Database (PLSD)

### P. Møller

#### PI to the PLSD

**Aim**

Compile existing prospective data on carriers of pathogenic MMR variants.

**Method**

Inclusion: 1) Demonstrated monoallelic germline carriers of pathogenic variant of either of the genes MLH1, MSH2, MSH6 or PMS2 listed in the InSiGHT database. 2) Determined to be at risk for Lynch Syndrome for any reason. 3) Inclusion point: First planned and carried out prospective colonoscopy. 4) One or more follow-up years.

Patient information: Age, gender, pathogenic variant, reporting centre, age and ICD9 diagnoses of all cancers (before, at or after inclusion), organs removed when.

Events scored: All prospectively diagnosed cancers after inclusion by ICD9 code and age at diagnosis. Age at death.

Information not yet analysed: polyps removed, stage at colorectal cancer and time since last colonoscopy.

For detailed protocol see https://ehtg.org/

**Results**

Incidences of cancer by age, genetic variant and gender. Survival after cancer. Results of intervention (international guidelines).

**Conclusion**

The reports migrate knowledge on Lynch syndrome from expert opinions based mainly on retrospective studies to assumption-free empirical observations in carriers subjected to follow-up according to accepted clinical guidelines. The interactive website www.PLSD.eu returning risk for any single case when indicating age, gender and gene is referred to for clinical use by InSiGHT and others.

## N40 Validated and updated risks for and survival after cancer by age and gender in Path_MSH2 carriers; a Prospective Lynch Syndrome Database (PLSD) Report

### P. Møller^1^, J. P. Plazzer^2^, T. Seppälä^3^, M. Dominguez-Valentin^4^, S. Nakken^5^, C. Engel^6^, S. Aretz^7^, H. K. Schackert^8^, W. Schmiegel^9^, N. Rahner^10^, M. von Knebel Doeberitz^11^, M. Löffler^12^, I. Bernstein^13^, L. Sunde^13^, M. Jenkins^14^, D. G. Evans^15^, F. Balaguer^16^, E. Holinski-Feder^17^, J. Burn^18^, L. Bertario^19^, A. Lindblom^20^, A. Della Valle^21^, R. H. Sijmons^22^, L. Katz^23^, W. H. de Vos tot Nederveen Cappel^24^, K. Heinimann^25^, N. Gluck^23^, C. A. Vaccaro^26^, F. Lopez-Koestner^27^, G. Martina Cavestro^19^, E. Hovig^5^, F. Macrae^28^, G. Möslein^29^, J. P. Mecklin^3^, J. Sampson^30^, G. Capella^16^

#### ^1^PI to the PLSD, Oslo University Hospital Norway and University of Witten-Herdecke Germany; ^2^InSiGHT database; ^3^Finland; ^4^PLSD curator Norway; ^5^Norway; ^6^German HNPCC consortium; ^7^University of Bonn; ^8^University of Dresden; ^9^University of Bochum; ^10^University of Düsseldorf; ^11^University of Heidelberg; ^12^University of Leipzig; ^13^Danish HNPCC Registry; ^14^Colon Cancer Family Registry: Australia, USA, Canada; ^15^Manchester, UK; ^16^Spain; ^17^Münich, Germany; ^18^Newcastle, UK; ^19^Italy; ^20^Sweden; ^21^Uruguay; ^22^Groningen, Holland; ^23^Israel; ^24^Leiden, Holland; ^25^Switzerland; ^26^Argentina; ^27^Chile; ^28^Melbourne, Australia; ^29^Germany; ^30^Cardiff, UK

**Correspondence:** P. Møller

**Aim**

Determine average risks for and survival after cancer in path_MSH2 carriers.

**Method**

Previously reported results were validated in an independent series of path_MSH2 carriers followed-up by colonoscopy. We combined results merging former and present series including only carriers with pathogenic class 4 or 5 variants listed in the InSiGHT database.

**Results**

The validation series including 11,684 observation years confirmed previously published cumulative risk for any cancer: at fifty years 35% compared to 37%, and at 75 years 79% compared to 80%. Combined series of carriers of path_MSH2 variants included 19,888 prospective observation years. Cumulative risk for cancer in specific organs or group of organs at 75 years in males/females were: Any cancer 73%/82%; colon_rectum 49%/45%; endometrium -/47%; ovaries -/17%; stomach_duodenum_bileduct_pancreas 19%/12%; ureter_kidney 17%/18%; urinary bladder 12%/8%; prostate 22%/-; breast -/14%; brain 7%/3%. Ten-year crude survival after cancer in different organs were: colon 94%; endometrium 86%; ovaries 81%; ureter_kidney 65%; urinary bladder 72%; prostate 51%, breast 74%; brain 18%. See www.PLSD.eu for risk determination in any single patient by age and gender.

**Conclusion**

The PLSD and InSiGHT databases are complementary: PLSD reports prospectively observed average risks and survival in carriers of variants determined to be pathogenic by InSiGHT.

## N41 Small bowel neoplasia detection in Lynch syndrome using video capsule endoscopy

### R. A. Zuppardo^1^, A. Contaldo^2^, C. Notaristefano^1^, M. Di Leo^3^, M. B. Principi^2^, A. Mannucci^1^, S. Rizzi^2^, M. Grazia Patricelli^4^, A. Russo Raucci^4^, P. A. Testoni^1^, G. M. Cavestro^1^

#### ^1^Gastroenterology and Gastrointestinal Endoscopy Unit, Division of Experimental Oncology, Vita-Salute San Raffaele University, IRCCS Ospedale San Raffaele Scientific Institute, Milan, Italy; ^2^Section of Gastroenterology, Department of Emergency and Organ Transplantation, University "Aldo Moro" of Bari, Italy; ^3^Digestive Endoscopy Unit, Division of Gastroenterology, Humanitas Clinical and Research Center, Department of Biomedical Sciences, Humanitas University, Milan, Italy; ^4^Division of Genetics and Cell Biology, Unit of Genomics for Human Disease Diagnosis, IRCCS Ospedale San Raffaele Scientific Institute, Milan, Italy

**Correspondence:** R. A. Zuppardo

**Aim**

Screening for small-bowel cancer (SBC) is not yet included in surveillance guidelines for LS. In 2016 Mallorca group advised may be appropriate in MSH2 and MLH1 mutation carriers, after 40 years. Aim of the study was to determine SBC incidence in asymptomatic LS patients by means of video capsule endoscopy (VCE).

**Method**

Two prospective VCE databases were retrospectively reviewed to identify consecutive asymptomatic LS patients, compared with a group of patients who underwent VCE for obscure gastrointestinal bleeding (OBS).

**Results**

25 LS patients and 280 OBS patients were enrolled by two Italian centers. In 91.5%, caecal visualization was achieved. SBC was detected in two LS patients and three OBS patients (p=0.06). The two groups have a significant statistically different mean age (SD): 41.3 ys± 14.0 ys in LS group and 62.9 ys ± 17.2 ys in OBS group. Besides SBC, LS patients and OBS patients have statistically significant difference in incidence of vascular lesion, angiectasia and minute polyps.

**Conclusion**

The prevalence of SBC in asymptomatic patients with LS was 8% vs 1.1%. Although the incidence of SMC did not reach statistical significance difference, a trend through statistically significant difference was observed and this suggests further multicentric studies are needed.

## N43 Hide and seek with hereditary cancer: testing the effectiveness and cost-effectiveness of implementation approaches for translating Lynch syndrome evidence into practice

### N. Taylor^1^, A. Morrow^1^, E. Hogden^1^, Y. J. Kang^1^, J. Steinberg^1^, K. Canfell^1^, M. Solomon^2^, J. Kench^2^, A. Gill^3^, T. Shaw^10^, N. Pachter^4,^ B. Parkinson^5^, L. Wolfenden^6^, G. Mitchell^7^, F. Macrae^8^, K. Tucker^9^

#### ^1^Cancer Research Division, Cancer Council NSW, Sydney, New south Wales, Australia; ^2^Royal Prince Alfred Hospital, Sydney, NSW, Australia; ^3^Royal North Shore Hospital, Sydney, NSW, Australia; ^4^Genetic Services of Western Australia, Australia; ^5^Macquarie University, Sydney, Australia; ^6^Newcastle University, Newcastle, Australia; ^7^University of British Columbia, Vancouver, Canada; ^8^Royal Melbourne Hospital, Melbourne, Australia; ^9^Prince of Wales Hospital, Sydney, Australia; ^10^University of Sydney, Australia

**Correspondence:** N. Taylor

**Aim**

Evidence indicates that hospitals face infrastructural, psychosocial and environmental barriers to detecting Lynch Syndrome (LS) patients. In Australia, less than half of all high-risk colorectal cancer (CRC) patients are being referred for LS genetic testing. This study aims to compare the effectiveness and cost-effectiveness of two implementation approaches for increasing the proportion of CRC patients with risk-appropriate completion of the LS testing and referral pathway.

**Method**

This randomised controlled trial will test the Theoretical Domains Framework Implementation approach against a non-theory-based implementation approach in eight large Australian hospitals. Site based healthcare professionals will be trained to lead the following process: 1) Baseline audits, 2) Form Implementation Teams, 3) Identify practice change behaviours, 4) Identify/confirm barriers to change, 5) Generate intervention strategies, 6) Support intervention implementation, 7) Evaluate practice/culture change. Theoretical and non-theoretical components are distinguished in 4-5.

**Results**

Progress to date of baseline data analysis will be presented. Plans for the analysis of health and economic outcomes of each implementation approach to be estimated using “POLICY1-Lynch” will be provided.

**Conclusion**

This will be a world first study to compare theory-based and non-theory based approaches to evidence translation in healthcare, and to incorporate these findings into existing microsimulation models to accurately assess implementation cost-effectiveness.

## N44 Genetic and clinical characteristics of registry-validated pedigrees of Lynch syndrome families in Slovenia – first report

### M. Krajc^1^, A. Blatnik^1^, G. Norčič^2^, S. Novaković^3^, V. Stegel^3^, J. Tavčar^1^, K. Strojnik^1^, V.Šetrajčič Dragoš^3^, G. Klančar^3^

#### ^1^Cancer Genetic Clinic, Institute of Oncology Ljubljana, Slovenia; ^2^Abdominal Surgery, University CLinical Center Ljubljana, Slovenia; ^3^Department of Molecular Diagnostics, Institute of Oncology Ljubljana, Slovenia

**Correspondence:** M. Krajc

**Aim**

The aim of this study was to assess genetic and clinical characteristics of Slovenian Lynch syndrome (LS) families, as such evaluation has not yet been performed for our population.

**Method**

We analyzed the results of genetic testing performed in 2008-2018 for probands fulfilling LS testing criteria. LS spectrum cancers identified in confirmed, obligate and assumed carriers of mismatch repair (MMR) gene mutations were verified in Slovenian cancer registry and analyzed according to site, age of onset, and genes involved.

**Results**

25 probands out of 251 tested carried a MMR mutation. 22 different mutations, 2 of which were recurrent, were identified. Mutation detection rate was 9.9%. 48% of probands harbored MLH1, 36% MSH2, 8% MSH6 and 8% PMS2 mutations. Of 120 cancers identified, 84 were colorectal (average age of onset: 42.2y) and 14 were endometrial carcinomas (average age of onset 52.4y).

**Conclusion**

We had very few referrals for LS testing in the 10-year period analyzed considering its prevalence in the population. LS is therefore likely to be drastically underdiagnosed in Slovenia. Screening of all colorectal and possibly endometrial cancers with immunohistochemical test should be performed in order to systematically identify LS families and offer them adequate treatment and familial risk assessment in the future.

## N45 High-definition white-light colonoscopy versus chromoendoscopy for surveillance of Lynch syndrome. A multicenter, randomized and controlled study (Endolynch Study)

### L. Rivero-Sánchez^1, 2, 3, 4^, C. Arnau^4^, F. Balaguer^1, 2, 3, 4, 5^, J. Herrero^6^, D. Remedios^6^, V. Alvarez^7^, E. Albéniz^8^, P. Calvo^8^, J. Gordillo^9^, I. Puig^10^, J. López Vicente^11^, A. Huerta^12^, M. López-Cerón^13^, I. Salces^13^, B. Peñas^14^, S. Parejo^14^, M. Herraiz^15^, A. Gimeno^16^, E. Saperas^17^, C. Alvarez^18^, L. Moreno^4^, C. Rodriguez de Miguel^13^, M. Diaz^4^, T. Ocaña^12^, L. Moreira^1, 2, 3, 4,5^, M. Cuatrecasas^1, 19^, S. Carballal^1, 2, 3, 4, 5^, A. Sánchez^1, 2, 3, 4, 5^, J. Llach^1, 2, 3^, M. Pellisé^1, 2, 3, 4, 5^

#### ^1^Hospital Clinic de Barcelona; ^2^Department of Gastroenterology; ^3^Digestive Endoscopy Unit; ^4^Institut d'Investigacions Biomediques August Pi i Sunyer (IDIBAPS), Barcelona, Spain; ^5^Center for Biomedical Research in the Hepatic and Digestive Diseases Network (CIBERehd). Barcelona, Spain; ^6^Complexo Hospitalario Universitario de Ourense, Department of Gastroenterology, Orense, Spain; ^7^Complejo Hospitalario de Pontevedra, Department of Gastroenterology, Pontevedra, Spain; ^8^Complejo Hospitalario de Navarra, Digestive System Service, Pamplona, Spain; ^9^Hospital de la Santa Creu i Sant Pau, Gastroenterology Unit, Barcelona, Spain; ^10^Althaia, Xarxa Assistencial Universitària de Manresa, Digestive System Service, Manresa, Spain; ^11^Hospital Universitario de Móstoles, Digestive System Service, Móstoles, Spain; ^12^Hospital Galdakao-Usansolo, Department of Gastroenterology, Galdakao, Spain; ^13^Hospital Universitario 12 de Octubre, Digestive System Service, Madrid, Spain; ^14^Hospital Ramón y Cajal, Department of Gastroenterology, Madrid, Spain; ^15^Clínica Universitaria de Navarra, Digestive System Service, Pamplona, Spain; ^16^Hospital Universitario de Canarias, Digestive System Service, Santa Cruz de Tenerife, Spain; ^17^Hospital General de Catalunya, Digestive System Service, Sant Cugat del Vallès, Spain; ^18^Hospital del Mar, Digestive System Service, Barcelona, Spain; ^19^Department of Pathology. Barcelona, Spain

**Correspondence:** L. Rivero-Sánchez

**Aim**

The use of pan-chromoendoscopy (CE) for surveillance in Lynch syndrome is currently recommended despite low evidence. We aimed to demonstrate that high-definition white-light endoscopy (WLE) is not inferior to CE for detection of adenomas.

**Method**

Patients with confirmed germline mismatch repair mutation were prospectively randomized 1:1 to WLE or CE performed by endoscopists devoted to high-risk conditions of colorectal cancer. The main outcome was the adenoma detection rate.

**Results**

256 patients (60% women; age 47±14y) were included in 14 centers. The detection rate of lesions in WLE versus CE group were: adenomas 28.1% versus 34.4% respectively (p=0.281), total polyps 50.0% versus 57.7% (p=0.004), proximal serrated lesions (SL) 10.2% versus 11.7% (p=0.689), sessile SL 5.5% versus 3.9%(p=0.554) and advanced adenomas 7.8% (4.3%-13.7%) versus 3.9% (1.6%-3.9%) (p=0.183) respectively. The mean (standard deviation) of lesions per patient for WLE versus CE were: adenomas 1.04 (1.37) versus 0.86 (1.04) (p=0.670), proximal SL 0.25 (0.56) versus 0.25 (0.61) (p=0.426), sessile SL 0.10 (0.31) versus 0.11 (0.67) (p=0.660), left-sided SL 0.21 (0.55) versus 0.53 (1.04) (p=0.002) respectively.The withdrawal time (minutes) for WLE and CE were 13.5 (5.63) versus 18.37 (7.57) (p<0.001) respectively.

**Conclusion**

In a scenario with expert endoscopists, WLE is an optimal and efficient endoscopic technique for surveillance of Lynch syndrome patients

## N46 The role of immunohistochemistry (IHC) testing in the tumor spectrum of the Lynch syndrome (LS)

### M. Marabelli^1^, P. R. Rafaniello^2^, M. Calvello^1^, I. Feroce^1^, M. Lazzeroni^1^, C. Ferrari^3^, A. Chiappa^3^, M. Barberis^2^, L. Bertario^1^, B. Bonanni^1^

#### ^1^Division of Cancer Prevention & Genetics, European Institute of Oncology, Milan, Italy; ^2^Division of Pathology, European Institute of Oncology, Milan, Italy; ^3^Unit of Innovative Surgical Techniques, European Institute of Oncology, Milan, Italy; University of Milan, Italy

**Correspondence:** M. Marabelli

**Aim**

To validate the performance of IHC testing of Mismatch Repair (MMR) proteins in patients with LS spectrum cancers.

**Method**

We analyzed MicroSatellite Instability (MSI) on 461 cancers (378 colorectal, 64 gynecologycal, 19 other sites). IHC analysis of MMR proteins was performed in all samples, irrespective of the MSI status. IHC results were classified as proficient-IHC (normal expression), deficient-IHC (loss of expression), borderline-IHC (“patchy” expression); borderline-IHC cases with MSI were classified as deficient-IHC.

Excluding samples with BRAF mutation or MLH1 promoter hypermetilation (MLH1-Hy), deficient-IHC cases were addressed to germline MMR gene testing.

**Results**

Fifty-three patients (11.5%) had deficient-IHC: 1 for all proteins, 1 for three proteins, 41 for two proteins (32 MLH1-PMS2, 9 MSH2-MSH6), 10 for 1 protein. IHC deficiency rate was significantly different among sites: 10% colorectal, 23% endometrial/ovarian cancer, 0% in other sites (p<0.001).

Twenty-five samples had BRAF mutation or MLH1-Hy. Twenty-eight patients, including 6 borderline-IHC, were addressed to genetic testing (16 ongoing) and mutations were found in 9 patients (4 in MLH1, 4 in MSH2 and 1 in MSH6), including one borderline-IHC with MSI.

**Conclusion**

We support the systematic evaluation of MMR proteins in colorectal and gynecological cancers to select patients with LS. MSI could be useful to manage borderline-IHC cases.

## N48 Molecular tumor testing in Lynch-like patients reveals de novo mosaic DNA mismatch repair gene pathogenic variants transmitted to offspring

### E. Guillerm^1^, H. Delhomelle^2^, M. Warcoin^1^, A. Palmyre^1^, M. Svrcek^2^, A. Bardier Dupas^1^, I. Sourrouille^2^, N. Janin^3^, M. Eyries^1^, V. Cusin^1^, F. Soubrier^1^, F. Coulet^1^, C. Colas^4^

#### ^1^Hôpital Universitaire Pitié Salpêtrière (Assistance Publique-Hôpitaux de Paris), Sorbone University, Paris, France; ^2^Hôpital Universitaire Saint Antoine (Assistance Publique-Hôpitaux de Paris), Sorbone University, Paris, France ; ^3^Cliniques Universitaires Saint-Luc, Brussels, Belgium ; ^4^Curie Institute, Paris, France

**Correspondence:** C. Colas

**Aim**

Lynch-like syndrome (LLS) patients have tumors with Microsatellite Instability but no germline variant in Mismatch Repair genes (MMR) or somatic methylation of the MLH1 promoter. Double somatic hits are the usual explanation for these cases. Our purpose was to find other explanations, such as mosaicism, that could explain LLS and have an impact on genetic counselling.

**Method**

We analysed the MMR genes in frozen tumor tissue samples by NGS for 28 LLS patients. When a tumoral variant was found, we performed a targeted re-examination of the germline NGS results with lower detection rates and targeted Sanger analysis in normal adjacent tissue DNA and lymphocytes DNA from offspring when available.

**Results**

Eight patients had double somatic hits in their tumors. Two patients had a germline de novo mosaic variant of MSH2 with low variant allele frequency (9% and less than 2%). Those variants were missed by NGS analysis in lymphocytes DNA. Their identification in tumors allowed a targeted NGS reanalysis. In both cases, these variants were found to be heterozygous in one of the offspring.

**Conclusion**

These mosaic cases confirm that identification of the mechanism that causes tumors in LLS is crucial for genetic counselling and guiding screening of patients and their relatives

## N49 A mouse model for proof of concept of a vaccine against Lynch syndrome-associated cancers

### M. Kloor^1^, M. Ozcan^2^, A. Ahadova^2^, Y. Yuan^3^, P. Bork^3^, S. Sei^4^, R. Shoemaker^4^, Ö. Gelincik^4^, S. Lipkin^4^, J. Gebert^2^, M. von Knebel Doeberitz^2^

#### ^1^Department of Applied Tumor Biology, University Hospital Heidelberg; ^2^University Hospital Heidelberg; ^3^European Molecular Biology Laboratory; ^4^National Cancer Institute (US)

**Correspondence:** M. Kloor

**Aim**

Microsatellite-unstable (MSI) cancers occurring in Lynch syndrome elicit pronounced immune responses directed against frameshift peptide (FSP) neoantigens. Our group could demonstrate the existence of shared FSP neoantigens in MSI cancer and detected spontaneous FSP-specific immune responses in affected patients. To further develop the concept of a cancer-preventive vaccine in Lynch syndrome, we aimed to establish a preclinical mouse model.

**Method**

A systematic database search was performed to identify coding microsatellites (cMS) and potential neoantigens in Lynch syndrome mice (Msh2flox/floxVpC+/+). After mutation analysis of murine tumors, most promising FSP neoantigens were evaluated for immunogenicity by ELISpot after vaccination of C57BL/6 mice.

**Results**

Four FSP neoantigens derived from common cMS mutations in the genes Nacad, Maz, Xirp1, and Senp6 elicited strong antigen-specific cellular and humoral immune responses. Based on the cMS mutation data, a vaccine with these four FSP neoantigens is predicted to cover about 75% of cancers in Lynch mice.

**Conclusion**

We have identified four immunogenic FSP neoantigens derived from commonly mutated cMS in murine Lynch syndrome colorectal cancers. This allows evaluating the concept of cancer-preventive neoantigen vaccines in mouse models of Lynch syndrome, including longitudinal monitoring of immune responses and combination with different adjuvants and chemoprevention approaches.

## N50 Age-related efficiency of BRAF V600E mutational testing for the exclusion of Lynch syndrome in MSI colorectal cancers

### H. Bläker^1^, A. Ahadova^2^, J. Chang-Claude^3^, A. Arnold^4^, M. von Knebel Doeberitz^2^, H. Brenner^3^, M. Hoffmeister^3^, M. Kloor^2^

#### ^1^Institute of Pathology, Charite Berlin, Germany; ^2^University Hospital Heidelberg, Germany; ^3^German Cancer Research Center, Germany; ^4^Charite Berlin, Germany

**Correspondence:** A. Ahadova

**Aim**

For distinguishing Lynch syndrome patients from sporadic microsatellite unstable (MSI) patients, BRAF V600E testing has become one of the most important tools. In order to analyze the discriminatory power of BRAF mutations in different age groups, we looked at the age distribution of BRAF mutations in MSI colorectal cancers.

**Method**

Age at diagnosis and BRAF mutation status were retrieved for unselected series of MSI colorectal cancers (n=151) from publicly available databases (DFCI) and the DACHS cohort.

**Results**

The prevalence of BRAF V600E mutations in MSI cancers strongly increased with age at diagnosis, with 87% of BRAF mutations occurring after the age of 65. There was no patient with a BRAF mutation under the age of 50, and the youngest patient with a BRAF mutation was 52 year old.

**Conclusion**

Our data demonstrate that BRAF mutation testing to exclude Lynch syndrome has very limited value in patients younger than 50, as the likelihood of detecting BRAF mutation in a patient under 50 is close to 0%. Reports of BRAF mutations in 1-2% of cancers from proven Lynch syndrome mutation carriers call into question the role of BRAF mutations as a bona-fide exclusion marker for Lynch syndrome.

## N51 A novel tool for quantitative analysis of microsatellite mutations and frameshift neoantigens

### A. Ballhausen^1^, M. Przybilla^2^, M. Jendrusch^2^, S. Krausert^2^, J. Janikovits^2^, A. Ahadova^2^, D. Heid^2^, S. Kalteis^2^, E. Pfaffendorf^2^, J. Gebert^2^, J. Krzykalla^3^, A. Benner^3^, M. von Knebel Doeberitz^2^, M. Kloor^2^

#### ^1^Department of Applied Tumor Biology, University Hospital Heidelberg, Germany; ^2^University Hospital Heidelberg, Germany; ^3^German Cancer Research Center, Germany

**Correspondence:** A. Ballhausen

**Aim**

Lynch syndrome cancers are caused by DNA mismatch repair (MMR) deficiency. MMR deficiency leads to microsatellite instability (MSI) and to a high mutational load. Insertion/deletion mutations (indels) of coding microsatellites are drivers of MSI cancer development and responsible for the accumulation of immunogenic frameshift neoantigens. Next-generation sequencing has a limited sensitivity for detecting such indels. We have developed a novel tool to provide a high-resolution map of the MSI cancer mutation and neoantigen landscape.

**Method**

The ‘qMSI’ algorithm processes fragment length analysis data, removing stutter band artifacts using a linear matrix. QMSI allows the quantification of the true allele frequency of mutations and the distinction of different mutation types that give rise to distinct frameshift neoantigens.

**Results**

Using qMSI for 40 target genes in MSI colorectal cancers (n=139) we demonstrate that most indels in MSI cancer are single-nucleotide deletions (77%) followed by two-nucleotide deletions and single-nucleotide insertions (21%). Neoantigen-inducing mutations were surprisingly similar across different MSI cancers.

**Conclusion**

The qMSI algorithm is a powerful tool to identify driver genes and mutational neoantigens in MSI cancer. The identification of shared, recurrent neoantigen-inducing mutations indicates that a vaccine for tumor prevention in Lynch syndrome is highly promising.

## N52 MMR deficiency is an early event in Lynch syndrome colorectal cancer pathogenesis

### A. Ahadova^1^, R. Gallon^3^, J. Gebert^2^, A. Ballhausen^2^, V. Endris^2^, M. Kirchner^2^, A. Stenzinger^2^, J. Burn^3^, M. von Knebel Doeberitz^2^, H. Bläker^4^, M. Kloor^2^

#### ^1^Department of Applied Tumor Biology, University Hospital Heidelberg, Germany; ^2^University Hospital Heidelberg, Germany; ^3^Newcastle University, UK; ^4^Charite Berlin, Germany

**Correspondence:** A. Ahadova

**Aim**

The onset of mismatch repair (MMR) deficiency in Lynch syndrome-associated tumors has been discussed to be a late event of pathogenesis. Since the time point of MMR deficiency onset and its consequences have a direct impact on the selection of suitable therapeutic and preventive measures, we aimed to reconstruct the sequence of mutational events in Lynch syndrome cancers.

**Method**

MMR protein expression and mutational signatures were analyzed to address the time point of MMR deficiency in Lynch syndrome adenomas and carcinomas from public databases and our own cohort.

**Results**

77% of Lynch syndrome adenomas (n=640) were MMR-deficient. Mutational signatures of MMR deficiency were reflected in canonical CRC gene mutations, demonstrating that 100% of KRAS and more than 60% of APC mutations likely occurred after the onset of MMR deficiency. A substantial proportion of Lynch syndrome-associated colorectal cancers lacked evidence of polypous growth. These tumors showed a distinct molecular pattern enriched for TP53 and CTNNB1 mutations.

**Conclusion**

There is more than one pathway of CRC development in Lynch syndrome. MMR deficiency commonly occurs early during Lynch colorectal carcinogenesis. Non-polypous cancers developing from MMR-deficient crypts may be missed by colonoscopy, strengthening the need for additional primary prevention measures in Lynch syndrome.

## N53 Discordant IHC MMR staining and MSI results in tumors of MSH6 mutation carriers

### A. S. van der Werf – ’t Lam^1^, M. S. Kan^1^, M. Suerink^1^, L. P. van Hest^2^, H. J. P. Gille^2^, A. Wagner^3^, C. Tops^1^, T. van Wezel^4^, H. Morreau^4^, S.ten Broeke^1^, M. Nielsen^1^

#### ^1^Department of Clinical Genetics, Leiden University Medical Centre, The Netherlands; ^2^Department of Clinical Genetics, VU Medical Center, Amsterdam, The Netherlands; ^3^Department of Clinical Genetics, Erasmus Medical Centre, Rotterdam, The Netherlands; ^4^Department of Pathology, Leiden University Medical Centre, The Netherland

**Correspondence:** M. Suerink

**Aim**

Diagnosing Lynch Syndrome caused by a MSH6 mutation can be challenging due to the relatively frequent occurrence of discordant immunohistochemistry staining (i.e. MSH6 positive staining) and microsatellite stable phenotype. The aim of this study is to describe to what extent discordant phenotypes occur in colorectal and endometrial carcinomas (CRC/EC) in MSH6 families.

**Method**

Data were collected from 192 MSH6 families with a confirmed segregating pathogenic germline variant ascertained from Dutch family cancer clinics.

**Results**

The data consists of 9719 family members and 838 proven mutation carriers. MSH6-mutation carriers with CRC or EC (n=306) were included in the study, accounting for 219 CRCs and 122 ECs. Of the tested tumors, discordant staining for MSH6 was reported in 10 out of 68 CRCs (14.7%) and 3 out of 26 ECs (11,5%). Six out of 62 CRCs (9.7%) and 5 out of 25 (20.0%) ECs appeared to be microsatellite stable. Fifteen germline MSH6 mutation carriers also displayed negative staining for MSH2 in addition to negative MSH6 staining, but did not harbor a germline MSH2 mutation.

**Conclusion**

Germline MSH6 mutation carriers can be missed using reflex IHC MMR testing as is currently standard in most western countries. MSH6 germline or tumor DNA analysis - preferably as part of a larger gene panel - should therefore be considered, especially in patients fulfilling the Bethesda criteria.

## N54 Characterisation of mismatch repair variants submitted to the International Mismatch Repair Consortium (IMRC)

### J. Reece^1^, D. Buchanan^1, 2^, G. Lee^1^, J. P. Plazzer^3^, K. Mahmood^4^, B. Pope^4^, M. Clendenning^2,^ A. Win^1, 5^, R. Haile^6^, G. Möslein^7^, F. Macrae^3, 8, 9^, M. Jenkins^1, 5^

#### ^1^Centre for Epidemiology and Biostatistics, Melbourne School of Population and Global Health, The University of Melbourne, Parkville, Victoria, Australia; ^2^Colorectal Oncogenomics Group, Department of Clinical Pathology, The University of Melbourne, Parkville, Victoria, Australia; ^3^Department of Medicine, The University of Melbourne, Parkville, Victoria, Australia; ^4^Melbourne Bioinformatics, The University of Melbourne, Parkville, Victoria, Australia; ^5^The University of Melbourne Centre for Cancer Research, Victorian Comprehensive Cancer Centre, Parkville, Victoria, Australia; ^6^Samuel Oschin Comprehensive Cancer Institute, Cedars-Sinai Medical Center, Los Angeles, California, USA; ^7^Helios St. Josefs-Hospital, Bochum-Linden, Germany; ^8^Genetic Medicine and Familial Cancer Centre, The Royal Melbourne Hospital, Parkville, Victoria, Australia; ^9^Department of Colorectal Medicine and Genetics, The Royal Melbourne Hospital, Parkville, Victoria, Australia

**Correspondence:** J. Reece

**Aim**

The IMRC contains data from 4624 Lynch families from 22 countries. We examined the geographical distribution of MMR mutations.

**Method**

Pedigree data includes: country, MMR mutation and cancer history. Frequency of each variant was calculated by geographical region.

**Results**

Of the 1578 unique MMR variants (MLH1=568, MSH2=582, MSH6=293, PMS2=135), the two most commonly reported variants were:

Gene: Variant (number of families with variant):

MLH1:

North America: c.350C>T (26), c.1852_1854del (22); Europe: c.1489dup (48), c.676C>T (28); South America: c.350C>T (6), c.1276C>T (6); Asia: c.381_453del (11), c.1852_1854del (4); Australasia: c.1852_1854del (12), c.350C>T (10)

MSH2:

North America: c.942+3A>T (88), c.(?_-125)_1076+?del (67) ; Europe: c.942+3A>T (130), c.1165C>T (25), c.1786_1788del (25); South America: c.942+3A>T (2), c.1077-?_1276+?del (2); Asia: c.1457_1460del (19), c.942+3A>T (5); Australasia: c.942+3A>T (16), c.2502_2508del (8)

MSH6:

North America: c.3261dup (18), c.2731C>T (12); Europe: c.3261dup (29), c.2731C>T (16)

South America: c.1519dup (2) ; Asia: c.3261dup (2) ; Australasia: c.3261dup (7), c.1571dup (5)

PMS2:

North America: c.137G>T (28), c.736_741delins11 (19); Europe: c.736_741delins11 (14), c.1882C>T (6)

South America: c.2186_2187del (2), c.2192_2196del (2) ; Asia: c.1572del (2), c.861_864del (2)

Australasia: c.736_741delins11 (11), c.989-296_1144+706del (4)

**Conclusion**

Some variants are frequently identified across geographical regions but heterogeneous distribution was found for other common variants. The IMRC has the potential to increase our understanding of the geographic distribution of Lynch syndrome.

## N56 Incorporating somatic sequencing into current molecular testing strategies for Lynch syndrome

### B. Desouza, G. Norbury, A. Kulkarni, D. Ruddy, V. Tripathi, L. Izatt, A. Shaw

#### Guy's Regional Genetics Service

**Correspondence:** B. Desouza

**Background**

UK guidelines recommend that all newly diagnosed colorectal cancers (CRCs) be screened for mismatch repair deficiency (MMR-D) that may be indicative of Lynch syndrome (LS). Current diagnostic approaches, will fail to detect MLH1 promoter hypermethylation or a germline mutation in approximately 60% of suspected LS cases. In most cases the diagnosis of LS can be excluded by somatic sequencing through the demonstration of double somatic mismatch repair (MMR) mutations.

**Method**

We have used our clinical data from over 1100 families to model costs for different diagnostic strategies for LS that integrate germline and somatic testing. Outcomes were correlated to family history category of either revised Bethesda guidelines or modified Amsterdam criteria.

**Results**

Modelling shows that for Bethesda families, performing concurrent germline and somatic testing would be more cost-effective than sequential germline and somatic testing (£523 vs. £940 per proband). For Amsterdam families, however, performing sequential testing would be more cost-effective than concurrent testing (£617 vs. £1256 per proband).

**Conclusion**

LS diagnostic strategies for CRC cases could be accelerated and simplified by concurrent germline and somatic testing. Moreover, our data suggests that this approach is more cost-effective than sequential testing in Bethesda families.

## N58 The cost of identifying Lynch syndrome carriers in Australia

### M. Dillon^1, 2^, M. A. Jenkins^2^, D. D. Buchanan^2^, D. A. Ouakrim^2^, L. Flander^2^

#### ^1^Aalto University, Finland; ^2^The University of Melbourne, Australia

**Correspondence:** M. Dillon

**Aim**

We estimated the cost of different screening strategies to identify Lynch syndrome (LS) mutation carriers in Australia.

**Method**

We used a microsimulation to model costs of DNA mismatch repair gene mutation testing for five target population subgroups: i) incident colorectal cancers (CRCs) diagnosed under age 50; ii) under age 70; iii) at any age; iv) unaffected people aged 20-50 years; and v) unaffected people aged 20-80 years. For the incident CRC subgroups, three strategies were considered: multi-gene panel testing; immunohistochemistry (IHC) followed by a multi-gene panel test; and IHC followed by MLH1 methylation testing and a multi-gene panel test. For the strategies targeting the general population (no CRC), only multi-gene panel testing was considered.

**Results**

IHC followed by panel testing yielded the lowest cost per mutation carrier identified at AU$2,529, AU$6,331, and AU$11,182 for the approaches targeting incident CRCs under age 50, 70 and any age, respectively. For the general population approaches, testing unaffected people aged 20-50 years was the cheapest option (AU$112,282 per carrier identified). Testing incident CRCs under age 50 identified the highest number of carriers (11,774 per 100,000 probands).

**Conclusion**

Testing incident CRC cases under age 50 years appears as the most effective and cheapest strategy to identify LS mutation carriers.

## N60 Lynch syndrome registries in Latin America

### A. Della Valle^1^, F. López-Kostner^2^, C. A. Vaccaro^3^, B. M. Rossi^4^, D. M. Carraro^5^, E. Palmero^6^, N. Manoukian Forones^7^, F. Spirandelli^8^, L. S. Lino-Silva^9,^, J. Sanchez del Monte^10^, J. Buleje^11^, C. M. Muñeton Peña^12^, Y. Sullcahuaman^13, 14^, K. Abe-Sandes^15^, I. Nascimento^16^, N. T. Rossi^17^, K. Alvarez^2^, F. Neffa^1^, T. Piñero^3, 18^, G. Tardin Torrezan^5^, S. Aguiar Junior^5^, C. Aparecida Marques Pimenta^7^, E. Spirandelli^8^, R. Fujita^11^, M. Torres Loarte^13, 14^, T. M. Bonfim Machado-Lopes^19^, T. Ferreria Bomfim-Palma^19^, L. Souza Mario Bueno^20^, S. T. dos Santos Nogueira^21^, C. Martin^17^, H. Galvão^6^, C. Dominguez-Barrera^22^, P. Wernhoff^23^, E. Hovig^23, 24^, P. Møller^23, 25, 26^, M. Dominguez-Valentin^23^

#### ^1^Hospital Fuerzas Armadas, Grupo Colaborativo Uruguayo. Investigación de Afecciones Oncológicas Hereditarias (GCU), Montevideo, Uruguay; ^2^Laboratorio de Oncología y Genética Molecular, Clínica Los Condes, Santiago, Chile; ^3^Hereditary Cancer Program (PROCANHE), Hospital Italiano, Buenos Aires, Argentina; ^4^Hospital Sirio Libanes, São Paulo, Brazil; ^5^AC Camargo Cancer Center, Sao Paulo, Brazil; ^6^Barretos Cancer Hospital, Barretos, Brazil; ^7^Gastroenterology Division, Universidade Federal de São Paulo (UNIFESP), São Paulo, Brazil; ^8^Servicio de Coloproctologia y Asesoria Genetica en Cancer, Hospital Español de Rosario, Rosario, Argentina; ^9^Departamento de Patologia, Instituto Nacional de Cancerologia, Mexico City, Mexico; ^10^Departamento de Endoscopia, Instituto Nacional de Cancerologia, Mexico City, Mexico; ^11^Centro de Genética y Biología Molecular, Instituto de Investigación, Facultad de Medicina Humana, Universidad de San Martín de Porres, Lima, Perú; ^12^Unidad de Genética Médica, Departamento de Pediatría, Facultad de Medicina, Universidad de Antioquia, Medellín, Colombia; ^13^Universidad Peruana de Ciencias Aplicadas, Lima, Peru; ^14^Instituto de Investigación Genomica, Lima Perú; ^15^Instituto de Ciências da Saúde/Universidade Federal da Bahia, Salvador, Brazil; ^16^Instituto de Ciência da Saúde e Núcleo de Oncologia da Bahia, Salvador, Brazil; ^17^Hospital Privado Universitario de Cordoba, Cordoba, Argentina; ^18^IMTIB-IU-Hospital Italiano de Buenos Aires, Argentina; ^19^Serviço de Oncogenética, Instituto de Ciências da Saúde, Universidade Federal da Bahia, Brazil; ^20^Complexo Hospital Universitário Professor Edgar Santos/Universidade Federal da Bahia, Bahia, Brasil; ^21^Oncoclin (Clínica oncológica), Amazonas Manaus, Brasil; ^22^Facultad de Enfermeria, Universidad Ricardo Palma, Lima, Peru; ^23^Department of Tumor Biology, Institute for Cancer Research, Oslo University Hospital, Oslo, Norway; ^24^Department of Informatics, University of Oslo, Oslo, Norway; ^25^Department of Medical Genetics, Oslo University Hospital, Norway; ^26^Department of Human Medicine, Universität Witten/Herdecke, Germany

**Correspondence:** M. Dominguez-Valentin

**Aim**

Despite significant advances in cancer genetics research in Latin America, the access to routine medical care for hereditary cancer patients is still limited.

Aim**:** To assess clinical resources for Lynch syndrome (LS) management across Latin American countries.

**Method**

International survey including selection criteria, clinical and genetic information was sent out to the 26 recently described LS programs from 10/33 countries of Latin America (Vaccaro et al. submitted).

**Results**

Amsterdam or Bethesda guidelines were mostly used to select patients for a tumor screening test and/or genetic MMR sequencing in 15/26 LS programs from public (n=7) or private (n=8) hospitals located in large urban areas from Argentina, Brazil, Chile, Colombia, Mexico, Peru and Uruguay. 717 LS carriers have been identified with a mean age of 42.5 years (range 32-50.9) and a mean of 3.7 follow-up years (range 1-9.6).

**Conclusion**

Several research projects and publications have been implemented, generating knowledge of MMR variants in these populations to bring additional awareness to medical professionals and public health leaders. Participation in PLSD and international collaborations have been initiated to support the implementation of genetic testing and research in most of the countries of Latin America.

## N62 Genetic and clinical features in Russian patients with Lynch syndrome

### D. Y. Pikunov, A. S. Tsukanov, S. I. Achkasov, V. P. Shubin, D. A. Semenov

#### State Scientific Centre of Coloproctology

**Correspondence:** D. Y. Pikunov

**Aim**

Up to 3% of all colorectal cancers are connected with Lynch syndrome (LS), which is caused by mutations in mismatch repair (MMR) genes. According to the literature the main manifestations of LS are tumors of right colon, endometrium, ovary, kidhey and ureter, stomach ets. at the age of <45.

**Method**

Between 2012 and 2017 ninety seven patients with primary tumors at the age of <45y.o. and/or with familial history were included in the study. All the tumors were analyzed for microsatellite instability (MSI). In patients with MSI the genes of MMR were examined.

**Results**

LS was diagnosed in thirty three (34%) out of 97 patients. Twenty of them (60%) had MLH1-gene mutation, 11 (34%) had MSH2-gene mutation, 2 (6%) – MSH6-gene mutation. The median age of primary tumor appearance in patients with LS was 38±7y.o. The primary tumor site was colorectum in 24 (73%) patients, uterus – in 8 (24%) patients, thyroid – in 1 (3%). Among the patients with colorectal cancer right colon lesions were registered in 5 (24%) cases and left colon – in 19 (76%).

**Conclusion**

In contrast to European patients, Russian patients with LS have MLH1-gene mutation in 60% cases, early-age appearance of colorectal cancer and preferable left-side lesions.

## N63 Clinical and molecular characterization of Lynch-like syndrome

### M. D. Picó^1^, R. Jover^2^, M. Giner^2^, M. Alustiza^2^, J. L. Soto^3^, A. Castillejo^3^, A. B. Sánchez^4^, A. Sánchez^5^, F. Balaguer^5^, L. Moreira^5^, A. Castells^5^, M. Pellise^5^, M. Carrillo-Palau^6^, T. Ramon y Cajal^7^, G. Llort^8^, C. Yagüe^8^, A. Lopez Fernandez^9^, J. Balmaña^9^, E. Martinez de Castro^10^, C. Alvarez^11^, X. Bessa^11^, J. Cubiella^12^, L. Rivas^12^, D. Rodríguez-Alcalde^13^, A. Dacal^14^, M. Herraiz^15^, C. Garau^16^, L. Bujanda^17^, L. Cid^18^, C. Povés^19^, M. Garzon^20^, A. Pizarro^20^, A. Gomez^21^, I. Salces^22^, M. Ponce^23^, E. Aguirre^24^, E. Saperas^25^, V. Piñol^26^

#### ^1^Gastroenterology, Hospital General Universitario de Elche, Elche, Alicante, Spain; ^2^Gastroenterology, Hospital General Universitario de Alicante, Alicante, Spain; ^3^Genetic. Hospital General Universitario de Elche, Alicante, Spain; ^4^Oncology. Hospital General Universitario de Elche, Alicante, Spain; ^5^Gastroenterology, Hospital Clinic Barcelona, Barcelona, Spain; ^6^Hospital Universitario de Canarias, Tenerife, Spain ; ^7^Hospital de la Santa Creu i Sant Pau, Barcelona, Spain ; ^8^Corporació Sanitària Parc Taulí. Sonsorci Sanitari Terrasa, Sabadell - Terrasa , Spain ; ^9^Hospital Vall d'Hebron, Barcelona, Barcelona, Spain ; ^10^Hospital Universitario Marques de Valdecilla, Santander, Spain ; ^11^Hospital del Mar, Barcelona, Barcelona, Spain ; ^12^Complexo Universitario Hospitalario de Ourense, Ourense, Spain ; ^13^Hospital Universitario de Móstoles, Mostoles, Spain ; ^14^Hospital Universitario Lucus Aungusti, Lugo, Spain ; ^15^Clinica Universitaria de Navarra, Navarra, Spain ; ^16^Hospital de Son Llàtzer, Palma de Mallorca, Spain ; ^17^Hospital de Donostia, Donostia, Spain; ^18^Hospital Álvaro Cunqueiro de Vigo, Vigo, Spain; ^19^Hospital clínico de San Carlos, Madrid, Spain; ^20^Hospital Universitario Virgen del Rocío, Sevilla, Spain; ^21^Hospital Universitario de Guadalajara, Guadalajara, Spain; ^22^Hospital 12 de Octubre, Madrid, Spain; ^23^Hospital Universitari i Politecnic de la Fe, Valencia, Spain; ^24^Hospital Quiron de Zaragoza, Zaragoza, Spain; ^25^Hospital General de Catalunya, Barcelona, Spain; ^26^Hospital Josep Trueta, Girona, Spain

**Correspondence:** M. D. Picó

**Aim**

The aim of this study is to know the clinical and molecular characteristics of LLS and to analyze if there are clinical, pathology or molecular characteristics that could suggest a hereditary or sporadic origin.

**Method**

This is a multicenter nation-wide study (25 Spanish hospitals). Patients were included when CRC tumors showed immunochemical loss of MSH2, MSH6, PMS2 or loss of MLH1 with BRAF-WT and/or no MLH1 methylation and absence of pathogenic mutation in these genes.

**Results**

Our study included 160 LLS patients. Loss of MLH1/PMS2 was found in 48% of CRC, loss of MSH2/MSH6 in 25%, loss of MSH6/PMS2 in 2%, isolated loss of MSH6, PMS2 and MSH2 was found in 11%, 9% and 2% respectively. In 3% of patients no gene loss of expression was found. 5 patients (3%) developed CRC during the follow up time since diagnosis, (median time of 7 years (SD 3.95)); 20 patients (12.5%) had personal history of non-CRC, and only 5 (3%) patients had LS-related cancer history.

**Conclusion**

In this LLS cohort, the largest until now, there are no clinical, molecular or pathological characteristics that could help distinguish between probably sporadic and hereditary patients. These results support the need of homogeneous follow-up for this group of patients.

## N64 Peritoneal and abdominal wall metastasis following colectomy in a patient with Lynch syndrome. Is it time to rethink the non-metastatic theory?

### P. C. Ambe^1^, D. Goedde^2^, G. Möslein^3^

#### ^1^Department of Visceral-, Minimally Invasive and Oncologic Surgery Marien Hospital Düsseldorf, Germany; ^2^Institute of Pathology and Molecular Pathology Helios, University Hospital Wuppertal, Germany ; ^3^Center for Hereditary Gastrointestinal Tumors Helios, University Hospital Wuppertal, Germany

**Correspondence:** P. C. Ambe

**Aim**

Hereditary non-polyposis colorectal cancer defines the development of colorectal cancer within the spectrum of presentation of Lynch syndrome. A major characteristic of CRC in Lynch individuals is the failure to metastasize despite the large tumor size. Herein we present a case of metastatic CRC in a patient with a pathogenic MSH2 / MSH 6 mutation.

**Method**

A 68 year old Caucasian male patient with a history of right nephrectomy 25 years after a uretheral cancer. Mismatch repair analysis confirmed MSI-H for MSH2 / MSH6. The patient now presented with a rectal cancer and to date he had not been recommended genetic testing. He underwent an anterior rectal resection with a protective loop ileostomy in December 2017 for colorectal cancer of the rectosigmoidal junction (pT3N0pV0pL0G2R0). An abdominal wall mass was found 10 months after surgery at the former ileostomy site during follow-up, which was completely excised with negative margins. Five months later, computed tomographic scans of the abdomen suspected recurrent metastasis including a peritoneal mass. Surgical exploration was performed.

**Results**

The abdominal wall mass was completely removed with negative margins. Equally, limited peritonectomy was performed during the second exploratory laparotomy. Histopathology confirmed the presence of metastasis from a colorectal cancer with loss of MSH 2 / MSH 6 proteins on immunohistochemistry. The patient was discussed at the interdisciplinary oncologic board after which adjuvant checkpoint inhibitor therapy was recommended However, the insurance was not willing to pay for this treatment.

**Conclusion**

Histopathologic features including loss of MSH 2 / MSH6 protein expression on immunohistochemistry in both the primary tumor as well as the metastatic lesions confirms the presence of metastatic seeding. This provides evidence for a metastasis of CRC in a patient with Lynch syndrome and disapproves the currently accepted non-metastatic theory. We conclude, that we cannot rely on the theory and are mandated to adhere to all principles of oncological surgery and also of stringent follow-up.

## N65 Etiology and characterization of Lynch-like syndrome patients

### M. Giner-Calabuig^1^, M. Juarez^1^, M. Alustiza-Fernández^1^, O. Murcia^2^, R. Jover^2^, X. Llor^3^, R. M. Xicola^3^

#### ^1^Fisabio-Isabial; ^2^Hospital General Universitario de Alicante Fisabio-Isabial; ^3^University of Yale

**Correspondence:** M. Giner-Calabuig

**Aim**

Lynch-like Syndrome patients are younger at diagnosis and had a higher prevalence of cancer in their families than individuals with sporadic cancers. These characteristics suggest the presence of an underlying hereditary condition.

The aim of this study is to characterize the molecular bases of LLS.

**Method**

We performed whole exome sequencing in a cohort of 27 LLS patients. We performed an analysis to identify rare likely pathogenic variants that could be predisposing to cancer. Only high-quality called variants, present with a population frequency <2.10-5 were included.

Based on the fact that the mutations in the MMR genes could be passenger mutations that drive further instability, a targeted analysis including a comprehensive list of DNA repair genes was also included.

We also performed tumor exome analysis from the matching samples to search for somatic hits.

**Results**

We identified 4 LLS patients with rare germinal variants in the following genes: AXIN1, PIWIL3, CD109, RECQL5 and GEN1. No somatic second hit was found in any of these genes. 2/8 cases where we could evaluate somatic events had a somatic mutation in one MMR gene and 1 showed LOH of the other copy. One tumor had a single mutation in a MMR gene and in one case I did not identify any somatic alterations.

**Conclusion**

Based on these results we hypothesize that there is a group of patients with predisposition to CRC due to a germinal variant in one allele that triggers genomic inestability. But there is also another group of patients where it could be due to a biallelic somatic mutation in MMR genes

## N66 The ICCon Australian database of mismatch repair variants

### F. Macrae^1^, J. P. Plazzer^1^, B. Thompson^2^, A. B. Spurdle^3^, G. Mitchell^4^, P. James^5^

#### ^1^Dept of Colorectal Medicine and Genetics, The Royal Melbourne Hospital, Australia; ^2^School of Population Health, University of Melbourne, Australia; ^3^QIMR Berghofer Medical Research Institute, Queensland, Australia; ^4^Chair ICCon Investigators; ^5^Head, Parkville Precinct Familial Cancer Clinics

**Correspondence:** F. Macrae

**Aim**

To systematically collect DNA mismatch repair variants identified by clinical testing in Australian families.

**Method**

Initial attempts through the HVP sourced variants from laboratories by streamlining with Laboratory Information Management Systems. Subsequently, a grant was awarded from the New South Wales Cancer Council to build a database of pathogenic (Class 4 and 5) variants identified in the cancer genes through the familial cancer clinics (FCCs); a collaboration across the clinics (ICCon) was formed to facilitate this.

**Results**

The ICCon database holds information about MMR gene pathogenic variants in adult carriers as follows: MLH1 124 (90 unique), MSH2 121 (94), MSH6 68 (50), PMS2 36 (25); totalling 349 (259). Ten discordant interpretations between clinics and/or InSiGHT’s classifications were resolved as part of the ICCon process. Importantly, clinical and other data to assist VUSs was accessible from the FCCs.

**Conclusion**

Sourcing variants via the FCCs has proved feasible. The ICCon database has contributed to variant interpretation internationally, including InSiGHT’s Variant Interpretation Committee and, in part, the PLSD. ICCon is working to achieve governance around transforming the variant database to a national registry, to permit changes in counselling, and clinical management, such as when new information emerges through contemporary experience or research.

## N67 Penetrance for carriers of a DNA mismatch repair gene specific variant

### A. K. Win^1, 2^, J. G. Dowty^1^, D. D. Buchanan^2, 3^, J. C. Reece^1^, G. Lee^1^, J. P. Plazzer^2^, G. Moslein^4^, R. W. Haile^5^, F. A. Macrae^6, 7^, I. M. Winship^2, 7^, M. A. Jenkins^1^, International Mismatch Repair Consortium (IMRC)

#### ^1^Centre for Epidemiology & Biostatistics, The University of Melbourne, Parkville, Victoria, Australia; ^2^Genetic Medicine, Royal Melbourne Hospital, Parkville, Victoria, Australia; ^3^Colorectal Oncogenomics Group, Genetic Epidemiology Laboratory, Department of Pathology, The University of Melbourne, Parkville, Victoria, Australia; ^4^Helios St. Josefs Hospital Bochum-Linden, Linden, Germany; ^5^Cedars-Sinai Medical Center, Los Angeles, CA, USA; ^6^Colorectal Medicine and Genetics, Royal Melbourne Hospital, Parkville, Victoria, Australia; ^7^Department of Medicine, The University of Melbourne, Parkville, Victoria, Australia

**Correspondence:** A. K. Win

**Aim**

Previous estimates of colorectal cancer risk for Lynch syndrome are averages over hundreds of different mutations in these genes. Reason for heterogeneity of cancer risk within specific variants in each gene is unknown.

**Method**

We estimated colorectal cancer risk for MSH2 c.942+3A>T variant using 234 families from the International Mismatch Repair Consortium. Age-specific cumulative risks (penetrance) and 95% confidence intervals were estimated using a modified segregation analysis with appropriate ascertainment conditioning and allowing for risk to vary between families by fitting a polygenic effect.

**Results**

The estimated average cumulative risks to age 70 years (95% confidence intervals), were 56% (38%-78%) for males carriers and 45% (28%-67%) for female carriers. However, the lifetime risks for different people were estimated to vary widely about these average risks (p=0.001). For carriers of this specific variant, 26% of males and 16% of females had colorectal cancer risk less than 20%; and 24% of males and 37% of females had risk greater than 70%.

**Conclusion**

Even for a specific variant in a DNA mismatch repair gene, there is a wide range of colorectal cancer risks. This is consistent with the existence of strong modifiers of risk, that if known, could be used to provide personalized risk of colorectal cancer for Lynch syndrome.

## N68 A multidisciplinary approach to familial pancreatic cancer enriches the proportion of patients with pancreatic cancer susceptibility

### R. A. Zuppardo^1^, A. Mannucci^1^, M. Reni^2^, M. Di Leo^3^, M. Grazia Patricelli^4^, A. Russo Raucci^4^, M. Falconi^5^, P. A. Testoni^1^, G. M. Cavestro^1^

#### ^1^Gastroenterology and Gastrointestinal Endoscopy, Division of Experimental Oncology, Vita-Salute San Raffaele University, IRCCS Ospedale San Raffaele Scientific Institute, Milan, Italy; ^2^Department of Medical Oncology, Vita-Salute San Raffaele University, IRCCS Ospedale San Raffaele Scientific Institute, Milan, Italy; ^3^Humanitas Clinical and Research Center, Department of Biomedical Sciences, Humanitas University, Milan, Italy; ^4^Division of Genetics and Cell Biology, Unit of Genomics for Human Disease Diagnosis, IRCCS Ospedale San Raffaele Scientific Institute, Milan, Italy; ^5^Division of Pancreatic Surgery, Department of Surgery, Vita-Salute San Raffaele University, IRCCS Ospedale San Raffaele Scientific Institute, Milan, Italy

**Correspondence:** G. M. Cavestro

**Aim**

Life-time risk of pancreatic cancer (PC) is 1.3%. Familial PC (FPC) have over 5% risk, due to family history and/or germline mutations. FPC accounts for 4-10% of all PCs, and germline mutations are detected in 5-10% of FPCs.

**Method**

Clinical and pathological data were retrieved during a single-session visit in gastroenterology and genetics from 2016 to 2017. FPC underwent either pancreatic endoscopic ultrasound (eUS) or magnetic resonance (MR) and Next Generation Sequencing analysis.

**Results**

57FPC were evaluated; 17 had a personal diagnosis of PC.

29(50,9%) had ≥2 relatives affected, of whom ≥1 was a first-degree relative (FDR); 11(37,9%) had PC.

11(19,3%) had ≥3 relatives affected (1 had PC). 6(10,5%) had Lynch Syndrome with ≥1 FDR (1 had PC). 2(3,5%) had hereditary pancreatitis and 9(15,8%) BRCA1/2 mutation with 1 FDR affected (5 had PC).

17(29,8%) were genetically confirmed: 6 LS (35,3%), 2 PRSS1 (11,8%), 6 BRCA2 (35,3%), 1 BRCA1 (5,9%), 2 PALB2 (11,8%). 8 showed a Variant of Unknown Significance (VUS). 21(36,8%) underwent eUS, revealing 8 PC, 3 intraductal mucinous neoplasias, 1 pseudopapillary lesion. 16(28,1%) underwent MR, revealing 7 CP, 1 IPMNs, and 3 cystadenoma.

**Conclusion**

A multidisciplinary approach enriches the proportion of patients with genetically confirmed FPC from 5-10% to about 30% of all FPC.

## N69 Interpretation of inheritable DNA variation: room for error across genetic services?

### M. Daly^1^, J. P. Plazzer^2,3^, F. Macrae^2, 3^

#### ^1^Melbourne Medical School, University of Melbourne, Melbourne, Victoria, Australia; ^2^Familial Cancer Centre, Royal Melbourne Hospital, Melbourne, Victoria, Australia; ^3^Department of Colorectal Medicine and Genetics, The Royal Melbourne Hospital, Melbourne, Victoria, Australia

**Correspondence:** F. Macrae

**Aim**

We aimed to evaluate the frequency of conflicts in interpretation of pathogenicity for gene variants in the mismatch repair genes MLH1, MSH2, MSH6 and PMS2 between InSiGHT’s Variant Interpretation Committee (VIC) and those provided by submissions from primary sources to ClinVar.

**Method**

Variant interpretation submissions for the four genes within ClinVar were compared and with those of the InSiGHT VIC. Factors that could account for the discordance were assessed including classification guidelines, evidence sources, research only interpretations.

**Results**

A total of 9,921 unique variant submissions were assessed. 584 interpretation conflicts were identified when compared to the VIC’s classifications. 98 of the conflicts were considered clinically significant. 5,862 variant interpretations have only one submitter. Methods of interpretation by submitters were heterogeneous and included clinical testing, research, and literature searching, accounting for much of the discordance.

**Conclusion**

Discordant interpretations between submitters represent opportunity for inconsistent counselling for families with the same variant, with potentially serious clinical consequences. Improvements in data sharing, increased support, coupled with increased awareness of the limitations of current generic methods for variant interpretation, and greater utilisation of expert panels who have access to comprehensive information and use clear gene specific criteria, are essential for optimal interpretation and safe clinical counselling.

## N72 CSTF2T and ACTB discern sporadic from FAP-associated colon carcinomas at various stages of carcinogenesis on the proteomic level

### T. Gemoll^1^, A. Masche^1^, U. J. Roblick^1^, F. Bader^1^, A. Unger^1^, S. Becker^2^, G. Möslein^3^, U. Hellmann^4^, H. Jörnvall^5^, H. P. Bruch^1^, G. Auer^2^, J. K. Habermann^1^

#### ^1^Department of Surgery, University Hospital Schleswig-Holstein, Campus Lübeck, Germany; ^2^Karolinska Biomic Center, Karolinska Institutet, Stockholm, Sweden; ^3^Department of Surgery, St. Josefs-Hospital Bochum Linden, Bochum, Germany; ^4^Ludwig Institute of Cancer Research, Uppsala, Sweden; ^5^Department of Medical Biochemistry and Biophysics, Karolinska Institutet, Stockholm, Sweden

**Correspondence:** T. Gemoll

**Aim**

Familial adenomatous polyposis (FAP) is an autosomal dominant inherited disease with a germline mutation of the APC gene. In spite of this specific genetic alteration early diagnosis in young patients without polyposis onset and lack of family history can be difficult and finally letal. Thus, additional sensitive diagnostics are required. We aimed at identifying and validating a protein expression signature in macroscopically unaffected colon mucosa that allows identifying genetic carriers of the FAP-syndrome.

**Method**

Protein profiling by 2-D gel electrophoresis was performed on samples obtained from 15 different patients (FAP, n=8; sporadic colorectal cancer, n=7). Analysis was performed for normal mucosa, adenoma, and carcinoma while comparing FAP-associated tissue with the sporadic counterpart. Analysis aimed at identifying proteins that were expressed in FAP tissue but not in the corresponding sporadic tissue, comparing particularly FAP associated normal mucosa versus sporadic normal mucosa. Target validation was performed by Western and by immunohistochemistry on clinical samples (n=189), respectively.

**Results**

A total of 47 proteins were present in all macroscopically unaffected FAP mucosa specimens but absent in sporadic normal mucosa. Comparing FAP polyps with sporadic colonic polyps revealed 49 polypeptides being present in FAP samples but absent in all sporadic polyps. Comparing three FAP carcinomas with seven sporadic colorectal carcinomas yielded 66 proteins with absence/ presence expression pattern. CSTF2T and ACTB were validated by Western Blot and immunohistochemistry in unaffected colon mucosa of FAP patients.

**Conclusion**

The data obtained demonstrate specific differences of FAP and sporadic colorectal disease on the protein expression level and could help to identify patients with FAP disease already in macroscopically “normal” colorectal mucosa.

## N73 The Danish HNPCC - register from 1991 to 2018

### I. Bernstein^1^, L. Lindberg^2^, L. Smith-Hansen^2^, L. Sunde^3, 4^

#### ^1^Surgical Gastroenterology Dept., Aalborg University Hospital, Denmark; ^2^The Danish HNPCC Register, Clinical Research Centre, Copenhagen University Hospital, Hvidovre, Denmark; ^3^Department of Clinical Genetics, Aarhus University Hospital, Aarhus, Denmark; ^4^Department of Biomedicine, Aarhus University, Aarhus, Denmark

**Correspondence:** I. Bernstein

**Aim**

Presenting the story of The Danish HNPCC-register and methods used for datacollection

**Method**

The Danish HNPCC register was established in 1991 as a private research register, later developing into a national database financed within the National Public Health care System. Epidemiological, clinical, and genomic data generated all over the country on 6.297 CRC families hereof 443 Lynch families are registered.

Initially paper-based reports were sent to and typed into the database. Later a model for electronic exchange of data between laboratories, departments and the register in an EC co-funded project to prevent cancer by optimizing screening, digitization of data transport and combining genotype-phenotype information, sufficiently usable and generic to be implemented in other countries were developed. As medical data are heterogeneous, focuses were on integration, development of classification systems and communication standards. Identified gaps and status of usability will be presented.

**Results**

Data in the HNPCC register belongs to the financing Capital Region and the multidisciplinary scientific societies providing data. To achieve commitment and ownership representatives off all parties are invited into the Scientific Board and Steering Comity of the register, where rules for ownership and data delivery are decided.

**Conclusion**

The Danish HNPCC-register is national and comprehensive, and researchers can request data via the Scientific Board.

## N74 Idiopathic pan-colonic varices found incidentally in a young patient with a hepatic flexure tumour: a rare cccurrence and a challenging surgical management

### O. AlZamzami, L. AlArfaj, H. AlOmran

#### KFSH-D

**Correspondence:** L. AlArfaj, H. AlOmran

**Aim**

Reporting the case of colon tumor in the presence of pancolonic varices and the surgical management we elected to do.

**Method**

Literature review

**Results**

Colonic varices is a rare entity and in majority of cases results from portal vein hypertension. It is even rarer when these lesions develop without an underlying hepatic or portal vein disease, termed, idiopathic colonic varices with less than 40 cases reported in the literature. Familial idiopathic colonic varices have also been described, where more than one family member is affected. These lesions could present an incidental finding, however, many cases presenting with lower GI bleeding were recognised in the literature, but no case was reported with a colonic tumor. Hereby we report a case of a 24 years old gentleman who presented with a history of acute abdominal pain and anemia. CT and Colonoscopy showed evidence of hepatic flexure mass, proved to be adenocarcinoma on histological examination, with an incidental finding of pancolonic varices. The patient has two relatives with pan-colonic varices on colonoscopy, but no history of colonic tumors. He underwent right hemicolectomy with uneventful recovery. To our knowledge, this is the first case reported with coexistence of extensive idiopathic colonic varices and colonic tumor.

## N75 Hereditary cancer predisposition syndromes: evaluation on the influence of personality in predictive genetic testing

### L. Moreno^1^, T. Ocaña^1^, A. Sánchez^1^, M. Salinas^2^, S. Iglesias^2^, A. Teulé^2^, J. M. Peri^1^, F. Balaguer^1^

#### ^1^Gastroenterology Department, Hospital Clínic de Barcelona, Centro de Investigación Biomédica en Red de Enfermedades Hepáticas y Digestivas (CIBERehd), Institut d’Investigacions Biomediques August Pi i Sunyer (IDIBAPS), Universitat de Barcelona, Spain ; ^2^Institut Català d’Oncologia, Hospital Duran i Reynals, Hospitalet de Llobregat, Spain

**Correspondence:** L. Moreno

**Aim**

Assess the psychological impact of genetic testing, evaluate changes in social life and behaviour, and estimate if personality influences the use of medical resources.

**Method**

Ten adults undergoing predictive genetic testing for cancer predisposition syndromes were included between January and March 2017. Demographic information, personality traits, psychological distress, behaviour in some daily activities and medical resources use were collected before testing and two months after results disclosure.

**Results**

High pre- and post-test psychological distress was associated to low education levels, having psychopathological history, pursuing testing for offspring, and being recruited at ICO (p<0.05). It was also associated with high negative affect, detachment, psychoticism and novelty seeking, and low reward dependence, self-directiveness, cooperativeness, and persistence (p<0.05). High post-test distress was also associated with having pre-test psychological distress (p<0.05). It would be important to know our counselees’ personality because it gives us the opportunity to know who to offer more support and how to personalize genetic counselling.

**Conclusion**

Our results suggest that there are some personality traits which can influence psychological distress in individuals undergoing predictive genetic testing. Further studies need to be performed in order to extrapolate these results to this particular population.

## N77 Correlation of immunohistochemical mismatch repair protein status in colorectal carcinoma endoscopic biopsy and resection specimens

### É. Ryan^2^, O. O’Brien^1^, B. Creavin^2^, M. E. Kelly^2^, H. M. Mohan^2^, R. Geraghty^1^, K. Sheahan^1^, D. C. Winter^2^

#### ^1^Department of Histopathology, St. Vincent’s University Hospital, Dublin, Ireland; ^2^Department of Surgery, St. Vincent’s University Hospital, Dublin, Ireland

**Correspondence:** É. Ryan

**Aim**

Microsatellite instability (MSI) is reflective of a deficient mismatch repair system (dMMR) and occurs in 15% of all colorectal carcinomas (CRC). This most frequently occurs due to sporadic or constitutional mutations in mismatch repair genes. Mismatch repair (MMR) status is often identified by immunohistochemistry (IHC) for mismatch repair proteins (MMRPs) on CRC resection specimens. IHC testing performed on endoscopic biopsy material may be as reliable as that performed on resected specimens. We aimed to evaluate the reliability of MMR IHC staining on preoperative CRC endoscopic biopsies.

**Method**

A retrospective search of our institution’s histopathology database was performed. Patients with CRC who had MMR IHC performed on both their preoperative endoscopic biopsy and surgical resection from 2010 - 2016 were included. Concordance of MMR staining between these specimens was assessed.

**Results**

53 patients had MMR IHC performed on both their preoperative endoscopic biopsy and resection specimens; 10 patients (18.87%) demonstrated loss of 1 or more MMRP on their endoscopic tumour biopsy. The remainder (81.13%) demonstrated preservation of staining for all MMRPs. There was 100% agreement in MMR IHC status between specimens in all cases (κ = 1.000, p < 0.000), with a sensitivity of 100% (95% confidence interval [CI]: 69.15-100] and specificity 100% (95% CI: 91.78-100) for detection of dMMR.

**Conclusion**

Endoscopic biopsies may provide a suitable source of tissue for MMR IHC analysis. This could allow a number of advantages to both clinicians and patients in the management of CRC.

## N78 In contrast to subjects with Lynch syndrome, the adenomatous polyps from subjects with sporadic MSI-high tumours have normal expression of MMR proteins

### A. Vilkin^1^, S. Morgenstern^2^, H. Weiss^2^, L. Haiiman Mantzur^2^, Y. Sternov^2^, Y. Goldberg^1^, I. Dotan^1^, Y. Niv^1^, Z. Levi^1^

#### ^1^Division of Gastroenterology, Rabin Medical Center, Petach Tikva, Israel; ^2^Department of Pathology, Rabin Medical Center, Petach Tikva, Israel

**Correspondence:** Z. Levi

**Aim**

Polyps from patients with Lynch Syndrome (LS) may show loss of expression of Mismatch Repair (MMR) proteins. Data about MMR expression in polyps from patients with sporadic MSI-high colorectal cancer is lacking. We investigated whether polyps form patients with sporadic MSI-High tumor may also show loss of MMR proteins expression.

**Method**

We performed IHC stain for four MMR proteins of adenomatous polyps from patients with sporadic MSH-high CRC vs. polyps from patients with LS. Sporadic MSI-high cancers were defined as tumors with loss of MLH1/PMS2 & BRAF V600E mutated.

**Results**

70 adenomatous polyps were analyzed: 22 from patients with sporadic MSH-High (81.8% women, median age 68.0 [IQR 61.7-86.2]) and 48 from LS patients (37.5% women, median age 48.5 [IQR 39.2-63.7]). Overall, none (0/22) polyps of the sporadic MSH-High group and 45.8 % (22/48) of the LS group showed loss of MMR protein stain (p<0.001). Of the LS group polyps, 41.5 %( 17/41) of polyps <10 mm and 71.4% (5/7) of polyps ≥10mm showed loss of protein expression p=0.145.

**Conclusion**

In contrast to LS, polyps from patients with sporadic MSI-high CRC do not show loss of MMR proteins. This may suggest that loss of MLH1 is a late event in the sporadic cases.

## N79 Immune microenvironment of colorectal carcinoma

### P. Janega^1, 2^, E. Gaal^2^, K. Giertlova^2^, J.Sedlak^3^, P. Babal^1, 2^

#### ^1^Medirex Group Academy, n.p.o., Trnava, Slovak Republic; ^2^Department of Pathology, Faculty of Medicine Comenius University in Bratislava, Slovakia; ^3^Cancer Research Institute, Slovak Academy of Sciences, Bratislava, Slovakia

**Correspondence:** P. Janega

**Aim**

The immune system plays crucial role in the development of the neoplastic diseases. Colorectal carcinoma is one of the most frequent oncological diseases with high mortality rate also in Slovak republic. Its development is the result of environmental, genetic and epigenetic changes accumulation leading to neoplastic transformation. Tumor-specific mutations manifest by neoantigens activating the immune system. The aim of the work was to evaluate the antitumor immune microenvironment in association of tumor grading.

**Method**

Archival surgical specimens of CRC were evaluated and graded according to the WHO criteria. Immunohistochemically detected CD4, CD8 and CD68 positive cells were evaluated morphometrically and expressed as % of the evaluated area.

**Results**

Neoplastic as well as the surrounding tissues were infiltrated by the three cell types in unchanged ratios, with predomonation of CD68+ histiocytes. With the increasing grade there was significant decrease of CD4+ and CD68+ cells and a clear decrease of CD8+ cells at the edge of significance, of infiltration of the tumor tissue. Changes in the peritumoral tissue infiltration were not significant.

**Conclusion**

Our findings support the idea of tumor suppressing activity of the anti tumor immunological response and that it plays an important role in progression of the neoplasm.

Supported by the APVV-14-0318 grant.

## N80 An international study of duodenal disease in MAP: incidence of polyposis and cancer

### L. E. Thomas^1^, A. Alonso Sanchez^2^, M. R. Aznárez^2^, A. Backman^3, 4^, J. Bjork^3, 4^, G. Capella^5^, S. K. Clark^6, 7^, C. Colas^8^, E. Dekker^9^, S. Dolwani^10^, Z. Ghorbanoghli^11^, M. Gonn^3, 4^, S. Gonzalez Romero^5^, F. J. Hes^12^, J. J. Hurley^13^, H. Jundi^1^, A. Latchford^6^, H. Leon Brito^2^, E. Meuser^1^, M. E. Mork^14^, M. Mort^1^, M. Navarro Garcia^5^, M. Neilsen^12^, Y. Parc^15^, M. T. Ricci^16^, J. C. Saurin^17^, K. van der Tuin^12^, H. Vasen^11^, E. Vilar^14, 18^, O. Vinet^17^, S. J. Walton^6, 7^, H. D. West^1^, J. R. Sampson^1^

#### ^1^Division of Cancer and Genetics, Cardiff University, School of Medicine, Cardiff, UK; ^2^GI Department, Complejo Hospitalario de Navarra, Pamplona, Spain; ^3^Hereditary Cancer Unit, Cancer Division, Karolinska University Hospital Stockholm, Sweden; ^4^Institution of Medicine, Solna, Karolinska Institutet, Stockholm, Sweden; ^5^Programa de Càncer Hereditari, Institut Català d'Oncologia, L'Hospitalet de Llobregat, Spain ; ^6^he Polyposis Registry, St Marks Hospital, Watford Road, Harrow, UK; ^7^Department of Surgery and Cancer, Faculty of Medicine, Imperial College, London, UK; ^8^Hopital Saint-Antoine AP-HP, Université Pierre et Marie Curie, Paris, France ; ^9^Department of Gastroenterology and Hepatology, Academic Medical Centre, Amsterdam, The Netherlands; ^10^Division of Population Medicine, Cardiff University School of Medicine, Cardiff, UK; ^11^Department of Gastroenterology & Hepatology, Leiden University Medical Centre, Leiden, The Netherlands; ^12^Leiden University Medical Center (LUMC), Department of Clinical Genetics, Leiden, The Netherlands; ^13^Dept. of Gastroenterology, Prince Charles Hospital, Merthyr Tydfil, UK; ^14^Clinical Cancer Genetics Program, UT MD Anderson Cancer Center, USA; ^15^Hopital Saint-Antoine AP-HP, Université Pierre et Marie Curie, Paris, France ; ^16^Unit of Hereditary Digestive Tract Tumors, Department of Surgery, Fondazione IRCCS Istituto Nazionale dei Tumori, Milan, Italy; ^17^Digestive Department, Edouard Herriot Hospital, Lyon, France; ^18^Department of Clinical Cancer Prevention, UT MD Anderson Cancer Center, USA

**Correspondence:** L. E. Thomas

**Aim**

Duodenal polyposis and cancer represent significant disease manifestations in patients with FAP and MAP. This study aims to determine the extent and incidence of duodenal disease in patients with MAP to establish whether upper GI surveillance recommendations developed for patients with FAP are also appropriate for MAP.

**Method**

A long-term prospective collaboration has been established. Demographic and genotype information and details of endoscopic surveillance and therapy has been collected on 394 MAP patients to date.

**Results**

63/394 had duodenal disease at index endoscopy (16%) at a median age of 54 years (range; 33-81): this was Spigelman stage I in 37 patients (58.7%), stage II in 12 (19%), stage III in 10 (15.9%), stage IV in 1 patient and three patients had cancer (4.8%). During 1417 follow up years, five further patients progressed to stage IV disease at a median age of 63 (range; 51-67) and one patient developed cancer.

**Conclusion**

Patients with MAP appear to develop fewer duodenal polyps at a more advanced age than is reported in FAP. Nonetheless, progression to advanced disease and cancer may occur despite surveillance. We are collecting prospective data that may inform development of a more appropriate surveillance strategy for upper GI disease in MAP.

## N81 Genomic and transcriptomic profiling of duodenal adenomas in Familial Adenomatous (FAP) and MUTYH-Associated Polyposis (MAP)

### E. Meuser^1^, K. Chang^2^, M. Mort^1^, J. J. Hurley^3^, K. Ashelford^1^, M. Naven^1^, N. Hawkes^3^, E. Short^1, 4, 5^, H. Jundi^1^, P. Georgiades^1^, M. W. Taggart^6^, L. Reyes-Uribe^2^, P. M. Lynch^7^, F. Neumann^8^, S. J. Walton^9, 10^, S. K. Clark^9, 10^, J. Sampson^1^, E. Vilar^2, 11^, L. E. Thomas^1^

#### ^1^Division of Cancer and Genetics, School of Medicine, Cardiff University, UK; ^2^Department of Clinical Cancer Prevention, UT MD Anderson Cancer Center, Houston, USA; ^3^Department of Gastroenterology, Cwm Taf University Health Board, Merthyr Tydfil, UK; ^4^Department of Histopathology, Cardiff, UK; ^5^Vale University Health Board, Cardiff, UK; ^6^Department of Pathology and Laboratory Medicine, UT MD Anderson Cancer Center, Houston, USA; ^7^Department of Gastroenterology, Hepatology and Nutrition, UT MD Anderson Cancer Center, Houston, USA; ^8^Science for Life Laboratory, Stockholm University, Sweden; ^9^The Polyposis Registry, St Marks Hospital, London, UK; ^10^Department of Surgery and Cancer, Faculty of Medicine, Imperial College, London, UK; ^11^Clinical Cancer Genetics Program, UT MD Anderson Cancer Center, Houston, USA

**Correspondence:** E. Meuser

**Aim**

Duodenal polyposis and cancer are important yet poorly understood causes of morbidity and mortality in FAP and MAP patients. We aimed to characterise the genomic and transcriptomic signatures associated with duodenal adenomas from patients with FAP and MAP, to better understand duodenal tumourigenesis in these hereditary disorders.

**Method**

A series of 67 samples from patients with a genetically confirmed diagnosis of FAP or MAP were subjected to whole transcriptome sequencing, consisting of 44 duodenal adenomas (FAP n=29, MAP n=15) and 23 duodenal normal mucosa (FAP n=15, MAP n=8). Outcomes were compared to exome sequencing data from 50 duodenal adenomas (FAP n=25, MAP n=25).

**Results**

We found distinct gene expression profiles in FAP and MAP duodenal adenomas which were absent from the respective normal mucosa. MAP adenomas harboured aberrations in RAS signalling and immune system stimulation, whilst evidence for dysregulation of prostanoid synthesis and NOTCH signalling were found in FAP adenomas. Whole exome analysis revealed that MAP duodenal adenomas carried more somatic mutations than FAP (p=0.0226). Recurrently mutated genes in duodenal adenomas included known drivers (APC, KRAS) and additional potential duodenal-specific tumour initiators.

**Conclusion**

The identification of commonly deregulated pathways contributes to our understanding of duodenal tumourigenesis in the context of FAP and MAP.

## N83 Surveillance recommendations for first-degree relatives of patients with unexplained multiple colorectal adenomas: a nationwide survey of UK regional genetic services

### B. Desouza, A. Elniel, N. Jakharia-Shah, G. Norbury, A. Kulkarni, D. Ruddy, V. Tripathi, A. Shaw , L. Izatt

#### Guy's Regional Genetics Service

**Correspondence:** B. Desouza

**Background**

Patients with multiple colorectal adenomas (MCRA; 10-100 adenomas cumulatively) without a known genetic cause are increasingly being diagnosed in the UK. Germline monoallelic APC or biallelic MUTYH mutations are not identified in the majority of patients. Possible explanations include; APC mosaicism, cryptic mutations, mutations in other polyposis genes, and polygenic inheritance. Some guidelines have recommended regular colorectal surveillance for first-degree relatives of this patient group, but currently there is no national UK guidance.

**Method**

We conducted a national survey of UK regional genetic services to explore management practices for first-degree relatives of patients with MCRA without a known genetic cause. A web based-survey was sent by email to the cancer genetic lead clinicians at the 24 regional genetics services. The survey was primarily designed to assess surveillance recommendations for first-degree relatives of MRCA patients, and to determine whether recommendations varied according to the total number of adenomas and age of onset. Testing criteria and genetic investigations were also assessed for patients with MCRA.

**Results**

National survey results are presented.

**Conclusion**

The survey aims to highlight variation in the management of this patient group and their first-degree relatives in the UK.

## N84 Mutations in MutYH gene among Russian patients with colorectal polyps

### Y. A. Shelygin, V. N. Kashnikov, A. M. Kuzminov, M. K. Toboeva, D. Y. Pikunov, V. P. Shubin

#### State Scientific Center of Coloproctology

**Aim**

MutYH-associated polyposis is one of the important inherited colorectal cancer syndromes. It is caused by germline mutations in the MutYH gene. Biallelic MutYH mutations are the genetic reason of an autosomal recessive mode of inheritance but we also observed risk of developing polyposis in monoallelic MutYH gene mutation carriers of some populations. The aim of this investigation was to study frequency of germline mutations in MutYH gene among Russian patients with different number of colorectal polyps

**Method**

Germline mutations in MutYH gene were detected by PCR, SSCP, Sanger sequencing and NGS among 19 patients with 100 and more colorectal polyps; 93 patients with 4-99 polyps and 150 healthy controls

**Results**

We found 11 germline mutations (8 biallelic and 3 monoallelic) in MutYH gene among 93 patients with 4-100 polyps and 2 mutations (1 biallelic and 1 heterozygous) in 19 patients with 100 and more colorectal polyps. We don’t found heterozygous mutations among 150 healthy controls

**Conclusion**

Frequency of germline mutations in MutYH gene among Russian patients with 4-99 and more than 100 colorectal polyps was 11,8% and 10,5% respectively

## N87 SELINA – clinical trial on lowering the risk of malignancies by optimizing selenium levels in females from families with hereditary breast cancer

### J. Lubinski

#### Department of Genetics and Pathology, Pomeranian Medical University in Szczecin and Read-Gene SA Poland

**Aim**

Blood selenium (Se) levels associated with significantly lower risk of cancers has been identified in Polish females from families with hereditary breast cancers (HBC). For BRCA1 mutation carriers: 70-89 μg/l at age <50 yrs (OR~12) and 95-120 μg/l at age ≥50 yrs (OR~4); for females without detected BRCA1 mutation: 98-108 μg/l (OR~5).

The main goal of SELINA is validation of hypothesis that optimization of Se level can decrease the risk of malignancies.

**Method**

7000 females (including 1200 BRCA1 carriers) from families with HBC and deficiency or excess of Se are qualified to one of the arms: “placebo”, observational, supplement (Sodium Selenite) or diet modification. Blood Se level will be measured and optimized during 5 yrs.

**Results**

Recruitment will be closed in 2018.

**Conclusion**

SELINA is the first trial aimed to decrease the risk of cancers by active control of blood selenium levels . Interested scientists are welcome for collaboration.

## N88 The national Lynch Syndrome Registry of Finland (LSRFi)

### T. Seppälä, K. Pylvänäinen, J. P. Mecklin

The nationwide Lynch Syndrome Registry of Finland (LSRFi) was founded in 1982 to organize endoscopic surveillance for high-risk families with colorectal cancer (CRC). To date, there are 298 families with confirmed pathogenic variants of mismatch repair (MMR) genes. Currently, LSRFi organises genetic counselling and predictive testing in research setting and co-ordinates endoscopic surveillance that takes place mostly in centralized public hospitals. Colonoscopy surveillance is offered from 25 years onwards, with 3-year interval for those with no prior cancer. LSRFi has access to national healthcare registries, such as registry for causes of death, parish registries and Finnish cancer registry.

About 3,000 individuals have undergone genetic testing, so far. In May 2018, there were total of 1,416 path_MMR carriers; 1,044 path_MLH1 (74%), 246 path_MSH2 (17%), 123 path_MSH6 (9%) and 3 path_PMS2 (0.2%). The mean age for live carriers was 53 years for path_MLH1, 53 years for path_MSH2, 60 years for path_MSH6 and 48 years for path_PMS2. In 2015, about two thirds of eligible children (age >18 years) of verified path_MMR carriers had undergone predictive testing. Adherence to offered surveillance is high, well over 90%. CRC incidence, stage and survival do not differ from other countries compared to independent prospective datasets in Europe.

**Acknowledgements**

On behalf of the Lynch Syndrome Registry of Finland (LSRFi)

## N90 Argentinean Lynch syndrome registry: experience from Rosario

### E. Spirandelli^1^, A. Naves^2^, S. Chialina^3^, F. Spirandelli^1^

#### ^1^Servicio de Coloproctologia y Asesoria Genetica, Hospital Español Rosario, Rosario, Argentina; ^2^Instituto de Histopatologia, Rosario, Argentina; ^3^Laboratorio Stem Rosario, Rosario, Argentina

**Aim**

There is still no national hereditary or familial cancer registers in Argentina. With the mission of improving detection, prevention and management of high risk cancer population in Rosario, with a population of 1.198.528 inhabitants, the Asociación Civil de Estudio, Tratamiento, Investigación de Enfermedades Heredo familiares de Rosario (ACETHIER) was established as a genetic reference center in 2005.

**Method**

Hospital Español is used to identify suspected Lynch syndrome (LS) families. The Amsterdam criteria (AMS) or Bethesda guidelines were mostly used to select cases for screening by immunohistochemistry (IHC) and/or microsatellite instability (MSI) analysis. Genetic testing was generally based on Sanger sequencing of *MLH1, MSH2, MSH6, PMS2* and/or *EPCAM.* By the advent of next generation sequencing (NGS), we are recently using 17- multigene panels including: *APC, BMPR1A, CDH1, CHEK2, MLH1, MSH2, MSH6, PMS2, MUTYH, POLD1, POLE, PTEN SMAD4, STK11, PT53*, EPCAM and GREM1(Ambry Genetics, USA). Patients are informed about their inclusion into the registry, which generally contained data on family history, clinical information, age at onset and results of DNA testing or tumour screening in the diagnosis of LS. Written informed consent was obtained from all patients during genetic counselling sessions.

**Results**

From our registry, 61 suspected families fulfilled AMS criteria or Bethesda guidelines. Seventeen families (28%) had MMR deficiency and underwent genetic MMR testing. *Path_ MLH1* variants was identified in 3 (21%) families, *path_ MSH2/EPCAM* variants in 11 (72%) families and *path_ PMS2* variants in 1 family (7%). LS carriers have been identified with a mean age of 37.5 years (range 18-57) and a mean of 13 follow-up years.

**Conclusion**

The *path_MSH2* variants are the most frequently identified in our registry and we provides support to set or improve LS genetic testing in South America. In addition, despite the small number of our registry, we described patients with a young age of onset and/or a positive family history of LS-associated cancers without an identified *path_MMR* variant, and may suggest the involvement of pathogenic variants in as yet undiscovered genes.

**Acknowledgements**

We would like to thanks Mev Dominguez-Valentin (Oslo University Hospital, Oslo, Norway), for her unconditional support and her effort, to be able to join all the research groups in Hereditary Colorectal Cancer from South America. She can lead this great Group, and we know that we will continue to grow.

## N91 Hereditary Cancer Program (ProCanHe): 21-years of experience at a referral registry in Argentina

### T. A. Piñero^1,2^, I. Herrando^2^, P. Kalfayan^2^, M. Gonzalez^2^, A. Ferro^2^, J. Santino^2^, R. Cajal^1^, D. Falconi^2^, G. Guerrero^2^, A. Verzura^2^, M. Riggi^2^, J. Church^3^, P. Peltomäki^4^, A. Martins^5^, W. Pavicic^2,4,6^, M. Dominguez^7^, C. Vaccaro^2^

#### ^1^Instituto de Medicina Traslacional e Ingeniería Biomédica(IMTIB)-CONICET-Instituto Universitario del Hospital Italiano-Hospital Italiano de Buenos Aires (HIBA), Argentina; ^2^ProCanHe, HIBA; ^3^Department of Colorectal Surgery, Cleveland Clinic Foundation, USA; ^4^Department of Medical Genetics, Biomedicum Helsinki, Finland; ^5^UFR de Médecine, France; ^6^IMBICE-CONICET, Bs.As., Argentina; ^7^Department of Tumor Biology Institute for Cancer Research, Oslo University, Norway

**Aim**

Registries in South America were initiated in the early 90´s with the help of Henry T. Lynch. The Programa de Cancer Hereditario (Pro.Can.He), is a multidisciplinary program established in 1996 at the Hospital Italiano, Argentina. The aim of the study is to update our 21-year experience to determine the applicability of genetic tests highlighting the most informative molecular findings in relation to Lynch syndrome mostly.

Materials and methods: Families undergoing genetic testing after genetic counselling between1996-2018 were included. Data were obtained from a prospective IRB approved database. Clinical-epidemiological and molecular variables were analysed. Genetic tests were carried out after a genetic counselling session and obtaining the informed consent of the patient.

**Molecular testing**

Until 2015, the search for variants was carried out by PCR and Sanger sequencing of exons and adjacent intronic regions of MLH1 and MSH2. Then, sequencing of *MLH1/MSH2/MSH6/PMS2/EPCAM* genes was performed by NGS and large rearrangements were detected by MLPA. The variants were classified according to international databases. Variants with uncertain or unreported clinical significance were analysed In-silico using the PolyPhen, SIFT and/or Human Splicing finder 3.0 software.

**Results**

A total of 83 families (49 fulfilled Amsterdam Criteria [AC] and 34 Bethesda Criteria [BC]) were analysed. Pathogenic variants were found in 26 out of 83 (31.3%) families, been 23 pathogenic and 3 likely pathogenic.

Splice site and large rearrangements represented 19.2% (5/26) and 11.5% (3/26) of the variants.23% (6/26) of them were originally described in this series and 1 was a founding mutation from Piedmont, Italy. Affected genes include MSH2, MLH1, MSH6 and PMS2 (12, 11, 2 and 1 cases respectively). Mutation detection rates in AC and BT families were 48.9% (N=24) and 5.9% (N=2), p<0.01. Among AC families, those with identified mutation had a lower median age of cancer on set and higher incidence of extra-CCR cancer than those without identified mutations. Additionally, we have also studied other genes in patients with different clinical conditions included in the registry.

We identified mutations in APC, MUTYH, BMPR1A, SMAD4, CDH1, BRCA1-2, CHEK2.

**Conclusion**

The multidisciplinary approach and the international collaborations allowed the correct implementation of the genetic tests. To our knowledge, this study is the first Characterization of AC families according to genetic tests in South America. This allowed the identification of AC families with different ages of onset and prevalence of extra-CRC cancers, as well as several significant variant not previously reported in international databases.

## N92 Chilean hereditary colorectal cancer registry: experience from Clinica Las Condes

### K. Alvarez, F. López-Köstner

#### Unidad de Coloproctología, Clínica Las Condes, Santiago, Chile

**Aim**

Considering the lack of genetic studies in our country and the benefits resulting from being able to differentiate between carrier and non-carrier individuals, in 2003 we applied for grant funds offered by the Chilean government (FONDECYT). During 2004-2006, this support enabled us to implement the MSI and IHC analyses in tumors, as well as the detection of point mutations in APC, MLH1 and MSH2 genes. In 2009, with the aim of increasing the mutation detection rate, genetic studies were supplemented with deletion/duplication analysis by MLPA for APC, MLH1, MSH2 and EPCAM genes, and the identification of point mutations in MUTYH, MSH6, PMS2, STK11, PTEN, SMAD4 and BMPR1A genes. Today, we have broadened the genetic studies into gene panels (Invitae, USA), mainly in those patients whose tumor studies do not allow us to define a candidate gene or when the definition of the hereditary syndrome becomes quite difficult.

**Methods**

Patients are referred to the program of hereditary colorectal cancer for evaluation. Those that meet criteria are included into the registry, which generally contained data on family history, clinical information, age at onset and results of DNA testing or tumor screening. Written informed consent was obtained from all patients during genetic counseling sessions.

**Results**

In our registry, we have an overall record of 221 suspected families (with 533 registered individuals), 107 are Lynch syndrome suspected families, 98 familial adenomatous polyposis, 11 Peutz-Jeghgers syndrome, 2 juvenil polyposis, 1 Cowden syndrome and 2 hyperplastic polyposis. In total, 88 families present a mutation or variant of uncertain significant in APC (41), MUTYH (3), MLH1 (21), MSH2 (7), MSH6 (1), PMS2 (3), EPCAM (2), STK11 (8), PTEN (1) and SMAD4 (1) genes. In those families with pathogenic or likely pathogenic mutations, we have studied 386 relatives, of which, 223 are carriers and 163 are no carriers. All families have received clinical recommendations based on the National Comprehensive Cancer Network (NCCN) guidelines. Interestingly, 25 mutations have not yet been described in other studies, clearly demonstrating the relevance of evaluating different racial/ethnic populations like ours, which include an admixture of Amerindian and European -mainly Spanish - populations.

**Conclusion**

Our work shows the success to integrate multidisciplinary professionals as coloproctologists, PhD in biological sciences (genetic counselor), nurses, medical doctors from various disciplines, and the constant support of a psycho-oncologist. We would like to highlight our last challenge, a pioneering initiative in Latin America, which consisted in the creation of a Course of genetic counseling in hereditary cancer aimed for health care professionals belonging to oncology units.

Acknowledgement: We would like to thanks Mev Dominguez-Valentin (Oslo University Hospital, Oslo, Norway), for her unconditional support and her effort.

## N93 Hereditary gastrointestinal cancer mutational registry In Uruguay

### P. Esperon^1,2^, F. Neffa^1^, N. Artagaveytia^1^, M. Sapone^1^, C. Vergara^1^, F. Carusso^1^, A. Della Valle^1^

#### ^1^Grupo Colaborativo Uruguayo; ^2^Facultad de Química. UDELAR Montevideo, Uruguay

**Introduction**

Since 1996, the Uruguayan Collaborative Group (UCG), a nonprofit organization is devoted to the registry, diagnosis, management and investigation of hereditary cancer. UGC is integrated by a multidisciplinary team of experts and represents in the country, a reference center for genetic counselling and risk assessment.

Objective: To present an updated Uruguayan mutation catalog for gastrointestinal (GI) hereditary cancer susceptibility

**Methodology**

The UCG registry is integrated by 1536 non-related families. 548 families (35%) are defined as GI-high risk population following the National Comprehensive Cancer Network 2018 guidelines. These families were classified as: Amsterdam I-II, Bethesda, Li Fraumeni, Peutz Jeghers, Familial Adenomatous Polyposis, *MUTYH*-Associated Polyposis, or Serrated polyposis syndrome. Selected probands for genetic testing signed informed consent prior to obtain saliva or blood samples.

Several DNA-analysis techniques were used over these 22 years, from Sanger sequencing alone (until 2010), Next Generation Sequencing of a group of genes and large rearrangements detection methods, to nowadays, panels of 30 genes.

**Results**

At present a total of 234 (43%) GI-high risk, non-related probands were tested and 63 families were diagnosed. We found 49 different mutations, classified according to ACMG as “Pathogenic” and distributed among the following genes: MLH1 (9), MSH2(11), PMS2(3), MSH6 (3), EPCAM(1), APC (11), STK11(2), NF1(1), FAN1(1), RAD51(1), SDHB(1), BMPR1A(1), MUTYH biallelic (3). A family carried a mutation class 4 (likely Pathogenic) in MLH1. In nine probands with a characteristic hereditary colon cancer phenotype, only MUTYH monoallelic mutations were found. An increasing number of variant of uncertain significance were found.

**Conclusion**

A research period of 22 years has unveiled the mutational spectrum of GI-high risk cancer of the Uruguayan population, allowing a broader vision regarding hereditary cancer profile in an understudied population. In spite of the large gene selection, only a few were involved in cancer predisposition. Lynch Syndrome, as expected, was the most frequent diagnosis, but with a relatively low pathogenic variant presentation.

**Acknowledgement**

Uruguayan Collaborative Group. Hospital Central de las Fuerzas Armadas, Montevideo, Uruguay.

Fundación Génesis Uruguay

## N94 Uruguayan hereditary breast and ovarian cancer syndrome registry: BRCA and non-BRCA pathogenic variants

### P. Esperon^1,2^, F. Neffa^1^, C. Acevedo^1^, G. Santander^1^, M. Sapone^1^, C. Vergara^1^, F. Carusso^1^, A. Della Valle^1^

#### ^1^Grupo Colaborativo Uruguayo; ^2^Facultad de Química, UDELAR Montevideo, Uruguay

**Introduction**

Mutations in *BRCA1* or *BRCA2* genes are considered the most prevalent cause of hereditary breast and ovarian cancer syndrome (HBOC), although other genes also explain this kind of affection. Since 2014, the Uruguayan Collaborative Group (UCG), a nonprofit organization is devoted to the registry, diagnosis, management and research of hereditary cancer, has been recruiting high-risk family groups with HBOC.

Objective: To report about pathogenic variants in BRCA and non-BRCA genes detected in Uruguayan high-risk for HBOC population.

**Methodology**

From the UCG registry, 592 non-related are defined as HBOC-high risk population following the National Comprehensive Cancer Network 2018 guidelines. Selected probands for genetic testing signed informed consent prior to obtain saliva or blood samples. Different approaches for searching gene mutations have been employed. At first, Next Generation Sequencing of BRCA1 and BRCA2, then large rearrangements detection methods were used, and lately multigene panels have been employed.

**Results**

330 (56%) HBOC-high risk, non-related probands were tested, 56 were found positives and 49 different pathogenic variants identified. BRCA1-2 accounted for 31 (66%) pathogenic mutations (14 BRCA1 and 17 BRCA2) while mutations in non-BRCA genes were: PALB2(3) ATM(1) CHEK2(3) BARD1(3), TP53(6), CHD1(1), NBN(1).

**Conclusion**

Even though only HBOC high risk probands were selected, a relatively high proportion of non-BRCA genes presented with pathogenic variants. Although multigene panels can give unexpected and uninformative results, when used with thoughtfulness, they can be a valuable tool capable of diagnose beyond the traditional boundaries of BRCA genes. Despite technological improvements, a high number of families with no molecular diagnosis still remains. Since the role of constitutive epimutations in cancer development can be underestimated, future approaches will include a methylation screening.

**Acknowledgement**

Fundación Génesis Uruguay. Laboratorio Genia Uruguay

